# Nationwide Registry-Based Analysis of Cancer Clustering Detects Strong Familial Occurrence of Kaposi Sarcoma

**DOI:** 10.1371/journal.pone.0055209

**Published:** 2013-01-24

**Authors:** Eevi Kaasinen, Mervi Aavikko, Pia Vahteristo, Toni Patama, Yilong Li, Silva Saarinen, Outi Kilpivaara, Esa Pitkänen, Paul Knekt, Maarit Laaksonen, Miia Artama, Rainer Lehtonen, Lauri A. Aaltonen, Eero Pukkala

**Affiliations:** 1 Genome-Scale Biology Research Program, and Department of Medical Genetics, Faculty of Medicine, University of Helsinki, Helsinki, Finland; 2 Haartman Institute, University of Helsinki, Helsinki, Finland; 3 Finnish Cancer Registry, Institute for Statistical and Epidemiological Cancer Research, Helsinki, Finland; 4 National Institute of Health and Welfare, Helsinki, Finland; 5 School of Health Sciences, University of Tampere, Tampere, Finland; The University of Texas M. D. Anderson Cancer Center, United States of America

## Abstract

Many cancer predisposition syndromes are rare or have incomplete penetrance, and traditional epidemiological tools are not well suited for their detection. Here we have used an approach that employs the entire population based data in the Finnish Cancer Registry (FCR) for analyzing familial aggregation of all types of cancer, in order to find evidence for previously unrecognized cancer susceptibility conditions. We performed a systematic clustering of 878,593 patients in FCR based on family name at birth, municipality of birth, and tumor type, diagnosed between years 1952 and 2011. We also estimated the familial occurrence of the tumor types using cluster score that reflects the proportion of patients belonging to the most significant clusters compared to all patients in Finland. The clustering effort identified 25,910 birth name-municipality based clusters representing 183 different tumor types characterized by topography and morphology. We produced information about familial occurrence of hundreds of tumor types, and many of the tumor types with high cluster score represented known cancer syndromes. Unexpectedly, Kaposi sarcoma (KS) also produced a very high score (cluster score 1.91, p-value <0.0001). We verified from population records that many of the KS patients forming the clusters were indeed close relatives, and identified one family with five affected individuals in two generations and several families with two first degree relatives. Our approach is unique in enabling systematic examination of a national epidemiological database to derive evidence of aberrant familial aggregation of all tumor types, both common and rare. It allowed effortless identification of families displaying features of both known as well as potentially novel cancer predisposition conditions, including striking familial aggregation of KS. Further work with high-throughput methods should elucidate the molecular basis of the potentially novel predisposition conditions found in this study.

## Introduction

Identification of inherited genetic factors that contribute to the development of cancer has been a subject of extensive research since the discovery of the first tumor susceptibility genes. Various approaches have been used to estimate the proportion of the inherited versus environmental factors in the etiology of different cancers. Twin studies have elucidated a large heritable fraction (26%–42%) for many common cancers, such as those of stomach, colon/rectum, breast, prostate, and lung [1]. Systematic registry-based analyses have shown a significantly increased risk for example for brain tumors, various lymphoma subtypes, and gastrointestinal neuroendocrine tumors in family members of affected individuals [2]–[5].

The earliest examples of cancer predisposing genes were identified in large cancer syndrome families. These families are rare and the contribution of these genes to the cancer burden as a whole is relatively small. For example, high-penetrance mutations in genes such as *MLH1*, *MSH2*, *APC*, and *MYH* account for only about 5% of all colorectal cancer cases [6]. However, identification of cancer predisposing genes has contributed significantly to our understanding of cancer biology, since they are often central also in the development of sporadic tumors.

The genetic background of the Finnish population (currently 5.4 million) is relatively well-studied and, even though considered relatively homogenous, has been shown to harbor substantial substructuring [7]. Ten distinct subpopulations of Finns have been characterized with high-density SNP genotyping suggesting multiple bottlenecks in the history of the Finnish population. After internal migration in the 1500s, population size of the wide inland areas of Finland grew in isolation because of long distances, and small villages formed genetically distinct subpopulations. Some degree of genetic admixture and migration due to urbanization started taking place after World War II. Single-origin mutations which have spread without negative effect on biological fitness have accounted for highly prevalent diseases in some of these isolated populations. This founder effect has enabled successful identification of genetic susceptibility to diseases in Finland (reviewed in [8]). As an example, the discovery of familial pituitary adenoma clusters in Northern Finland led to the identification of *aryl hydrocarbon receptor interacting protein* (*AIP*) defects in patients with pituitary adenoma predisposition [9]. Mutations in *AIP* originally identified in Finland have subsequently been found from similar patients worldwide [10].

The Finnish Cancer Registry (FCR) is a nation-wide database covering currently more than one million patients, and practically all incident cancers and cancer deaths in Finland since 1953 [11]. Physicians, hospitals, and pathology and hematology laboratories send cancer notifications to FCR. This reporting was made obligatory by law in 1961. Thus, FCR has also a link to sample information, such as archival tissue blocks, for all the patients that have been examined in pathology laboratories. Information about family history and family relationships is not recorded in the cancer notifications, but identification of patients’ relatives is possible through the National Population Registry (NPR) and the Parish Registries (PRs). PRs have kept records of citizens since 1580 until NPR was established. The personal identity code (PIC) system was implemented in Finland in the 1960s, and PIC is used as the identification key also in FCR for all patients except for those who died before 1967. Both FCR and NPR have an excellent coverage and high quality data [11], [12].

Conventional epidemiological tools are poorly suited to search for evidence of cancer predisposition, in particular if the conditions in question are uncommon. Here we describe an effective method that utilizes the extensive patient data in FCR, and information about family name at birth and municipality of birth in NPR, to systematically identify familial aggregation of all different tumor types on a nation-wide scale. The method enables identification of families suitable for sample collection and further research of the underlying genetic predisposition. Similar approach in a smaller scale has earlier been exploited in a search for prostate cancer families in Finland [13]. In this study, we apply our method to cluster all available patients in FCR. We calculate statistical significance for each cluster, and produce information about familial occurrence of hundreds of tumor types. We describe in detail a strong familial aggregation and uneven geographical distribution of Kaposi sarcoma (KS) in Finland. Furthermore, we report several families with two first degree relatives and a striking family of five cases with KS.

## Materials and Methods

### Ethical Considerations

The research registry and data processing procedures were evaluated and approved by the Ministry of Social Affairs and Health in Finland.

### Patient Data in the Finnish Cancer Registry

We considered 1,175,040 neoplasms registered in 1953–2011 in FCR. This included 212,685 cases of certain commonly registered precancerous lesions such as basal cell carcinoma of skin and polycythemia vera (registered since 1969). Currently, the disease classification in FCR is based on the ICD-oncology, 3rd edition.

### Systematic Clustering

Prior to clustering, the tumor types were classified further to topography and morphology groups (Tables S1 and S2). Tumor types with at least two patients in FCR were included, resulting in 846 combinations of topography and morphology (Table S3). To obtain information on patients’ family names at birth and municipalities of birth, we linked the 878,593 patients with PIC to NPR.

For each tumor type, systematic clustering was performed based on municipality of birth and family name at birth (MN-clusters). The observed number of patients (O) with each tumor type in FCR was calculated in a stratum defined by municipality (M), family name (N), sex, and year of birth. The proportion of persons in each M, N, sex, and year of birth category in the entire population of same gender and year of birth was calculated from the same version of NPR database that was linked with FCR patients. When that proportion was multiplied with the total sex and birth-year specific number of cancer cases of the cancer type in question in the NPR-linked subset of FCR, the expected number of cases (E) in each stratum was obtained. The stratum-specific observed and expected numbers were added up over the gender and birth year categories. O/E ratios were calculated for each MN category, and their 95% confidence intervals were defined assuming a Poisson distribution of observed numbers.

### Estimation of Familial Occurrence with Cluster Score

To estimate the familial occurrence of various tumor types, cluster score was calculated. For each tumor type *i*, we selected clusters with the lower limit of confidence interval (CI-low) greater than or equal to α, and calculated the number of patients (X*_i_*_α_) in these clusters.

We define the cluster score of tumor type *i* to be X*_i_*_α_ divided by the total number of patients with the same tumor type (Y*_i_*) per 100,000 persons in Finland. For example, cluster score with α = 10 for Kaposi sarcoma was calculated as following (X = 19 and Y = 537 from Table 1):
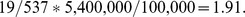



**Table 1:** Tumor types showing the strongest evidence for familial occurrence.

Topography	Morphology	Cluster score	p-value*	Number of patients in FCR	Number of clusters**	Number of patients in clusters**	Suggestive predisposition gene
central nervous system	hemangioblastoma	4.98	2.77E−14	141	5	13	*VHL*
thyroid gland	medullary carcinoma	3.55	7.86E−21	335	9	22	*RET*
Skin	Kaposi sarcoma	1.91	2.05E−13	537	9	19	
Pancreas	neuroendocrine carcinoma	1.40	2.75E−06	386	5	10	*MEN1, RET*
Kidney	nephroblastoma	1.26	5.98E−06	428	5	10	*WT1*
small intestine	neuroendocrine carcinoma	0.98	1.19E−09	1154	10	21	
hematopoetic and reticuloendothelialsystem	myelosclerosis	0.69	2.75E−09	2186	14	28	
vulva and vagina	squamous cell neoplasms and carcinoma	0.63	1.37E−10	3092	17	36	
mesothelium	mesothelioma	0.62	3.49E−06	1750	10	20	
Breast	mucinous and mucinous cystic tumor	0.57	2.45E−07	2564	13	27	
thyroid gland	papillary adenocarcinoma	0.56	2.77E−20	7863	41	82	
central nervous system	neurofibroma	0.55	1.02E−05	2062	9	21	*NF1*
central nervous system	neoplasm malignant	0.51	1.89E−05	2346	11	22	
hematopoetic and reticuloendothelialsystem	polycythemia vera	0.50	6.07E−05	2173	10	20	
hematopoetic and reticuloendothelialsystem	chronic lymphatic leukemia, b-cell	0.48	3.37E−13	7478	31	66	
Kidney	neoplasm malignant	0.47	1.20E−05	3015	13	26	
lymph node	Hodgkin lymphoma	0.46	2.77E−09	5499	22	47	
esophagus	squamous cell neoplasms and carcinoma	0.45	8.37E−09	5514	23	46	
Prostate	epithelial neoplasm and carcinoma	0.44	2.82E−05	3187	13	26	
Lip	squamous cell neoplasms and carcinoma	0.35	1.04E−05	7167	22	46	

* Poisson distribution, two-sided test with 95% confidence level, p-values adjusted with FDR for multiple comparisons** CI-low ≥1.

To estimate whether X*_i_*_α_/Y*_i_* was different than expected ratio Y_i_*X_α_/Y (where X_α_/Y is the respective ratio over all tumor types in Finland; 0.0029 for α = 10), two-sided Poisson test was calculated with 95% confidence level. The p-values were adjusted for multiple test comparisons with false discovery rate method [14].

### Confirmation of Kinship in the Clustered Kaposi Sarcoma Cases

Relatedness of patients in KS clusters was confirmed using information from NPR and PRs. NPR includes a link to parents for persons born since 1950s and who had not died before the PIC system was implemented in the 1960s. For the rest of the patients, we utilized PRs to identify first degree relatives and their children. PRs were also utilized for more thorough genealogy research. The relatives of the clustered KS cases were traced back at least three generations. To identify additional affected cases in the families, the cancer histories were checked from FCR for individuals identified through genealogy.

### Geographical Illustration of Kaposi Sarcoma in Finland

The O/E ratios for Kaposi sarcoma cases in each municipality of birth were calculated as described previously for MN-clusters, and illustrated using the small-area mapping method developed in FCR [15]. The twenty-one largest cities in Finland, including Vyborg (currently part of Russia), are shown as circles with a diameter relative to E, and with the color shading indicating the O/E ratio in that city. The remaining map area illustrates the floating averages of the O/E ratios of the neighboring municipalities, excluding the largest cities. The map was based on borders of Finland before year 1940 because a large proportion of the KS patients were born before that.

### Determination of HHV8 Seroprevalence in Finland

To estimate Human herpesvirus 8 (HHV8) infection rate in Finland, serological testing was performed in age and sex matched serum samples of 200 Eastern and 200 Western Finns obtained from the National Public Health Institute’s Health 2000 cohort. The serological analyses were done at HUSLAB (Helsinki, Finland) with direct HHV8 IgG antibody Enzyme Linked ImmunoSorbent Assay.

## Results

We clustered 878,593 cases in FCR to assess the familial aggregation of various tumor types defined by topography and morphology. The clustering was performed based on tumor type, municipality of birth, and family name at birth (MN-clusters). As a result, we identified 25,910 MN-clusters representing 183 combinations of topography and morphology (see Table S3 for distribution of cases). All the clusters fulfilled the criterion that the number of cases in the cluster was higher than the expected number based on the distribution of family names and birth municipalities in Finland, with CI-low≥1.

We also sought to estimate the tumor types that showed strongest evidence for familial occurrence, and for that we calculated cluster score for tumor types that produced significant MN-clusters. The clusters with the strongest confidence, CI-low≥10, were considered, encompassing 4.7% of all MN-clusters and representing 169 tumor types (Table S3). This corresponds to 0.3% of the patients in FCR.

Tumor types and respective cluster scores with FDR adjusted p-values<0.0001 are displayed in Table 1. Cluster scores and p-values for all tumor types are listed in Table S3. Most of the top scoring tumor types indicated rare cancer predisposition syndromes. The most frequently clustered tumor type was hemangioblastoma, a common finding in patients with the Von Hippel-Lindau syndrome (VHL [MIM #193300]) (cluster score 4.98). The second most frequently clustered tumor type was medullary thyroid carcinoma indicative of patients with Multiple Endocrine Neoplasia type 2 (MEN2A [#171400] and MEN2B [#162300]) (cluster score 3.55). Among the most common tumor types showing strong evidence for familial occurrence were papillary thyroid adenocarcinoma (cluster score 0.56), chronic lymphatic leukemia (cluster score 0.48), and squamous cell carcinoma of the lip (cluster score 0.35), all with over 7000 registered patients in FCR (Table 1).

### Kaposi Sarcoma

**Figure 1:**
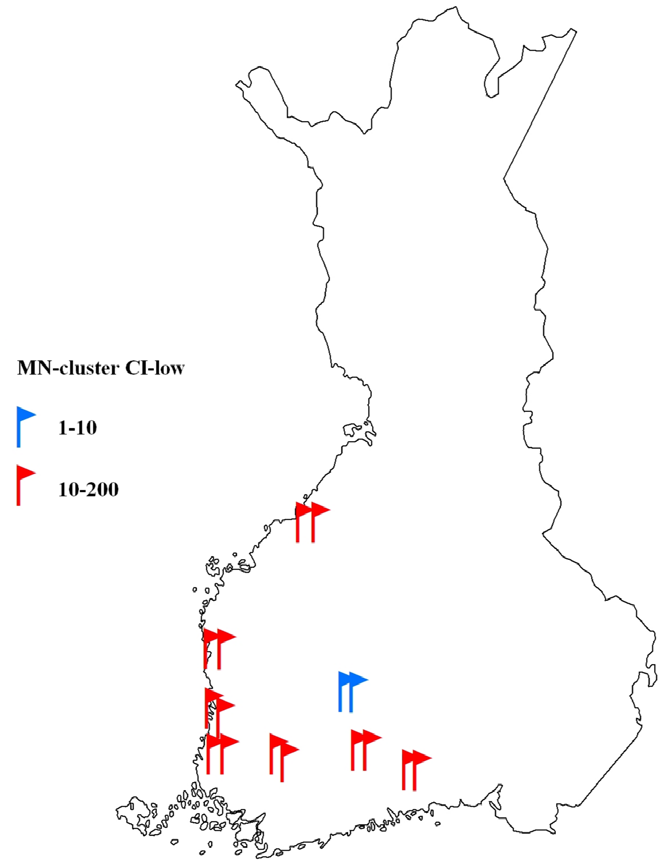
Familial Kaposi sarcoma (KS) cases in Finland; each flag marks a confirmed familial KS patient belonging to a cluster of municipality of birth and family name at birth (MN-cluster). Red and blue colors represent the lower confidence limit (CI-low) of MN-clusters. The borders of Finland are based on those before year 1940.

**Figure 2:**
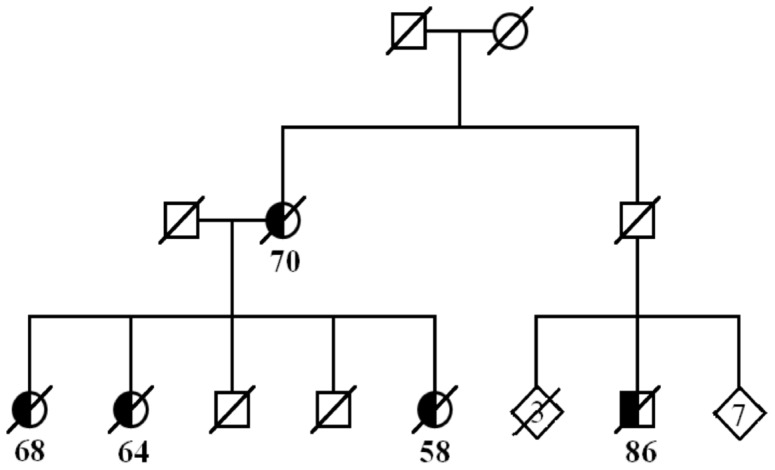
Expansion of a key pedigree; a family of five patients with Kaposi sarcoma (KS) from Eastern Finland. The age at diagnosis of KS is shown for each affected individual.

Familial occurrence of KS, the third most frequently clustered tumor type (cluster score 1.91), was further studied through genealogy as an example of the procedure to be followed with promising clusters in the systematic search. Out of the 23 KS patients in the MN-clusters, 16 (70%) were shown to be first-degree relatives within the cluster, and 14 of these belonged to clusters with CI-low≥10 (Fig. 1). The genealogy revealed a family of five affected individuals with KS in two generations (Fig. 2). Two family members belonged to the same MN-cluster, and a third sibling and an affected cousin were connected to the family through genealogy work. We were able to confirm the mother’s KS diagnosis (she had died in 1962) from the radiotherapy records after it was verbally reported by her daughter.

**Figure 3:**
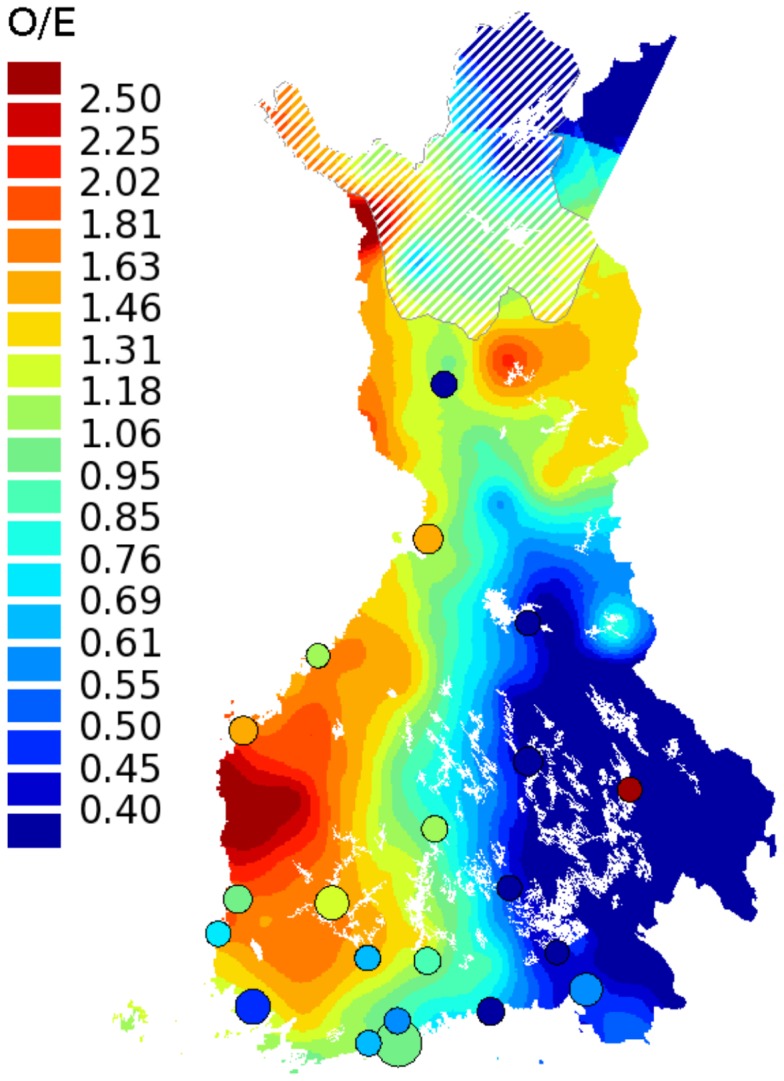
The observed/expected (O/E) ratios of Kaposi sarcoma (KS) incidence according to municipality of birth. Persons born in rural Western Finland have much higher incidence of KS than persons born in Eastern Finland. Twenty-one largest cities are indicated by circles. The white lining is superimposed on the colors of the sparsely populated areas in Northern Finland (less than 1 inhabitant per km^2^). The borders of Finland are based on those before year 1940.

The geographical distribution of KS incidence according to municipality of birth of the 537 KS patients in FCR showed a strikingly uneven pattern (Fig. 3). Patients accumulate almost entirely to the rural areas of Western Finland. Another aggregation of KS patients is seen in Northeastern Finland, while Eastern Finland displays much less KS cases than expected by chance. Interestingly, the family of five patients (Fig. 2) originates from Eastern Finland, whereas most of the other ascertained familial cases originate from Western Finland (Fig. 1).

Human herpesvirus 8, also known as Kaposi sarcoma-associated herpesvirus, is a well-known etiologic factor for KS. To study whether the uneven clustering of KS cases resulted from the uneven distribution of HHV8 infections in Finland, we studied the seroprevalence of HHV8 in 400 individuals from Eastern and Western Finland. Four out of 400 individuals (1%) were HHV8 positive. Two of them originated from Eastern Finland and two from Western Finland, suggesting similar seroprevalence of HHV8 in Eastern and Western Finland. However, the numbers are far too small for a proper conclusion on possible geographical variation of HHV8 infection in Finland.

## Discussion

Here we have used an approach to systematically analyze familial aggregation of all tumor types in nation-wide registry-based data. Unlike most traditional epidemiological tools, our approach was able to derive evidence for familial occurrence of both common and rare tumor types. Tumor types showing the greatest potential for familial occurrence were those that are mainly seen in rare cancer predisposition syndrome patients - a proof of concept that the method works well in estimating familial occurrence. Unexpectedly, one of the top scoring tumor types was Kaposi sarcoma with strong familial aggregation and uneven geographical distribution. We were able to identify seven families with two first-degree relatives with KS, and a family with five KS cases, which to our knowledge is the third largest reported KS family worldwide [16], [17].

The described clustering method is designed to identify cancer cases with similar phenotype and common family name at birth, thus the method is able to detect also potential relatives outside the nuclear family. The method is also applicable to other registered diseases than cancer. Primary requirement is to obtain information about family names and municipalities of patients, while population-scaled data is needed for calculation of statistical significance. Populations with enough diversity in family names are suitable for similar clustering efforts and allow the formation of statistically significant clusters. Due to the fact that family name in Finland is usually inherited from father, the birth name-municipality based clustering method is restricted to finding family links only from the paternal side.

In this study, we were able to cover significantly more cases and more detailed tumor types compared to previous familial clustering efforts, such as those conducted in Icelandic [18] or Utah population databases [19], [20]. One limitation of our effort is the lack of such extensive genealogy data for the studied individuals as in the studies in Iceland or Utah. However, the diversity of family names in Finland (∼100,000) and relatively little migration before World War II – the time period when most of the cancer cases in FCR were born – warrants our search for familial occurrence with MN-clustering. In our example case of KS, we were able to ensure familial relations through NPR and PRs for 70% of the cases in MN-clusters, and 88% of these clusters were of high statistical significance with the lower confidence limit greater than ten. Thus, calculating the statistical significance of clustering, and choosing clusters with the high O/E and CI-low values obviously increase the probability of identifying true relatives.

We concentrated on the most significant MN-clusters to estimate the familial occurrence of the tumor types using cluster score. Top two tumor types indicated known rare cancer syndromes, namely Von Hippel-Lindau syndrome and Multiple Endocrine Neoplasia type 2, suggesting that our approach is feasible in identifying families with high-penetrance cancer predisposition. One of the most interesting phenotypes with a high cluster score was KS, which showed high tendency to cluster with a frequency exceeding, for example, that seen in Wilm’s tumor. Another phenotype among the top tumor types without known genetic etiology was neuroendocrine carcinoma of small intestine, for which high familial risk has been described earlier [3], [21].

Twin studies and systematic registry-based studies of all cancers have been restricted to demonstrate the hereditary effect concerning the most common cancer sites. In our study, the strongest evidence for familial occurrence among the most common tumor types was shown for papillary thyroid adenocarcinoma, chronic lymphatic leukemia, and squamous cell carcinoma of the lip, each with over 7000 registered patients in FCR. The very same cancer sites, although without detailed morphology definitions, showed significant relatedness also in distant familial relationships (no closer than first cousins) in Utah [20].

KS is a prevalent tumor type especially among HIV-infected patients and in sub-Saharan Africa [22]. HHV8 is a well-established etiologic factor for KS, and it is well acknowledged that patients suffering from immunosuppressive conditions have higher risk for KS [23], [24]. A few reports exist on familial occurrence of KS [16], [17], [25]–[28]. Accumulating evidence suggests genetic background as a predisposing factor to KS. A number of studies have associated certain human leukocyte alleles, and genetic variants in other host immunity related genes, with predisposition to KS [29]–[35]. However, no conclusive mutations predisposing to familial KS have thus far been reported. KS is a vascular tumor with an average incidence of 0.1–0.2 per 100,000 person-years in Finland in 1963–2010, adjusted for age to the World Standard Population. The incidence of KS according to municipality of birth varied strongly in Finland. Most KS patients were born near the west coast, leaving Eastern Finland with significantly fewer KS cases (Fig. 3). It is well reported, in a number of seroepidemiological studies, that HHV8 infection rates differ geographically [36]. Whether the west-east difference in the incidence is due to prevalence of the virus or local founder effect remains to be studied more carefully. Our preliminary serological analyses showed that the HHV8 infection is rare in Finland, and does not seem to be differentially distributed. HHV8 seroprevalence has not been studied in Finland earlier, whereas Finland’s neighboring countries, Sweden in the west and Russia in the east, show seroprevalences of 10–20% and <10%, respectively [22]. The risk of HHV8 infection is higher among family members of classic KS patients, and person-to-person transmission has been confirmed by the observation that in almost all cases the same viral genotype was shared within the family [37], [38]. While intrafamilial transmission of HHV8 probably plays a role in the familial occurrence of KS in our data, it cannot explain in itself the number of KS patients as high as five within a single family without additional susceptibility to develop tumors.

It is likely that some high-penetrance tumor predisposing genes – particularly those of recessive inheritance and those that do not display easily recognizable syndromic features – remain unidentified. Poorly characterized combinations of multiple germline variants with moderate penetrance are likely to explain some proportion of the hereditary cancer burden. While such genetic susceptibility factors have previously been difficult to study, the molecular tools available for hereditary cancer gene identification have improved dramatically in recent years. Next-generation sequencing has highlighted the utility of very small numbers of carefully chosen affected individuals in genetic analyses [35], [39], [40]. Our approach is useful in deriving the most valuable specimens from the whole population for analyses of new cancer susceptibility conditions, including archival tissue blocks that can be utilized for generation of high-throughput DNA data for predisposition variant detection.

## Supporting Information

Table S1 **ICD-O-3 based morphology code groups used in the birth name-municipality based clustering.**
(DOCX)

Table S2 **ICD-O-3 based topography code groups used in the birth name-municipality based clustering.**
(DOCX)

Table S3 **Topography and morphology combinations in the birth name-municipality based clustering, distribution of patients, and cluster scores.**
(DOCX)
