# Supplementary material for: Nationwide Registry-Based Analysis of Cancer Clustering Detects Strong Familial Occurrence of Kaposi Sarcoma
Source: PLoS One. 2013 Jan 24;8(1):e55209. doi: 10.1371/journal.pone.0055209 (PMC3554690; doi:10.1371/journal.pone.0055209)
Supplement: Table S1 — ICD-O-3 based morphology code groups used in the birth name-municipality based clustering. (DOCX) [file pone.0055209.s001.docx]

**Table S1. ICD-O-3 based morphology code groups used in the birth name-municipality based clustering.**

| Morphology (ICD-O-3)* | Behaviour (ICD-O-3) | Morphology Group | Group Definition |
| --- | --- | --- | --- |
| 8550 | 3 | 1 | acinar cell carcinoma |
| 9801 | 3 | 2 | acute leukemia |
| 9805 | 3 | 2 | acute leukemia |
| 9840 | 3 | 3 | acute myeloid leukemia |
| 9861 | 3 | 3 | acute myeloid leukemia |
| 9866 | 3 | 3 | acute myeloid leukemia |
| 9867 | 3 | 3 | acute myeloid leukemia |
| 9871 | 3 | 3 | acute myeloid leukemia |
| 9872 | 3 | 3 | acute myeloid leukemia |
| 9873 | 3 | 3 | acute myeloid leukemia |
| 9874 | 3 | 3 | acute myeloid leukemia |
| 9891 | 3 | 3 | acute myeloid leukemia |
| 9896 | 3 | 3 | acute myeloid leukemia |
| 9910 | 3 | 3 | acute myeloid leukemia |
| 9920 | 3 | 3 | acute myeloid leukemia |
| 9931 | 3 | 3 | acute myeloid leukemia |
| 8140 | 3 | 4 | adenocarcinoma |
| 8140 | 2 | 4 | adenocarcinoma |
| 8140 | 1 | 4 | adenocarcinoma |
| 8142 | 3 | 4 | adenocarcinoma |
| 8144 | 3 | 4 | adenocarcinoma |
| 8145 | 3 | 4 | adenocarcinoma |
| 8147 | 3 | 4 | adenocarcinoma |
| 8180 | 3 | 4 | adenocarcinoma |
| 8201 | 3 | 4 | adenocarcinoma |
| 8210 | 3 | 4 | adenocarcinoma |
| 8211 | 3 | 4 | adenocarcinoma |
| 8245 | 3 | 4 | adenocarcinoma |
| 8250 | 3 | 4 | adenocarcinoma |
| 8253 | 3 | 4 | adenocarcinoma |
| 8255 | 3 | 4 | adenocarcinoma |
| 8290 | 3 | 4 | adenocarcinoma |
| 8323 | 3 | 4 | adenocarcinoma |
| 8370 | 3 | 4 | adenocarcinoma |
| 8525 | 3 | 4 | adenocarcinoma |
| 8530 | 3 | 4 | adenocarcinoma |
| 8570 | 3 | 4 | adenocarcinoma |
| 8576 | 3 | 4 | adenocarcinoma |
| 8200 | 3 | 5 | adenoid cystic carcinoma |
| 8560 | 3 | 6 | adenosquamous carcinoma |
| 8390 | 3 | 7 | adnexal and skin appendage neoplasm |
| 8400 | 3 | 7 | adnexal and skin appendage neoplasm |
| 8401 | 3 | 7 | adnexal and skin appendage neoplasm |
| 8402 | 3 | 7 | adnexal and skin appendage neoplasm |
| 8407 | 3 | 7 | adnexal and skin appendage neoplasm |
| 8408 | 3 | 7 | adnexal and skin appendage neoplasm |
| 8409 | 3 | 7 | adnexal and skin appendage neoplasm |
| 8410 | 3 | 7 | adnexal and skin appendage neoplasm |
| 8413 | 3 | 7 | adnexal and skin appendage neoplasm |
| 9310 | 0 | 8 | ameloblastoma |
| 9310 | 3 | 8 | ameloblastoma |
| 9384 | 1 | 9 | astrocytoma |
| 9400 | 3 | 9 | astrocytoma |
| 9401 | 3 | 9 | astrocytoma |
| 9411 | 3 | 9 | astrocytoma |
| 9412 | 1 | 9 | astrocytoma |
| 9420 | 3 | 9 | astrocytoma |
| 9421 | 1 | 9 | astrocytoma |
| 9424 | 3 | 9 | astrocytoma |
| 9430 | 3 | 9 | astrocytoma |
| 8090 | 3 | 10 | basal cell carcinoma |
| 8093 | 3 | 10 | basal cell carcinoma |
| 8094 | 3 | 10 | basal cell carcinoma |
| 8095 | 3 | 10 | basal cell carcinoma |
| 8098 | 3 | 10 | basal cell carcinoma |
| 8102 | 3 | 10 | basal cell carcinoma |
| 8123 | 3 | 11 | basaloid carcinoma |
| 8124 | 3 | 11 | basaloid carcinoma |
| 9823 | 3 | 12 | chronic lymphatic leukemia, b-cell |
| 9940 | 3 | 12 | chronic lymphatic leukemia, b-cell |
| 9687 | 3 | 13 | burkitt lymphoma |
| 9826 | 3 | 13 | burkitt lymphoma |
| 8950 | 3 | 14 | carcinosarcoma |
| 8951 | 3 | 14 | carcinosarcoma |
| 8980 | 3 | 14 | carcinosarcoma |
| 8160 | 3 | 15 | cholangiocarcinoma |
| 8162 | 3 | 15 | cholangiocarcinoma |
| 9220 | 3 | 16 | chondrosarcoma |
| 9220 | 0 | 16 | chondrosarcoma |
| 9230 | 3 | 16 | chondrosarcoma |
| 9231 | 3 | 16 | chondrosarcoma |
| 9370 | 3 | 17 | chordoma |
| 9390 | 1 | 18 | choroid plexus papilloma and carcinoma |
| 9390 | 0 | 18 | choroid plexus papilloma and carcinoma |
| 9390 | 3 | 18 | choroid plexus papilloma and carcinoma |
| 9863 | 3 | 19 | chronic myeloid leukemia |
| 9875 | 3 | 19 | chronic myeloid leukemia |
| 9876 | 3 | 19 | chronic myeloid leukemia |
| 9945 | 3 | 20 | chronic myeloproliferative disorder |
| 9948 | 3 | 20 | chronic myeloproliferative disorder |
| 9960 | 3 | 20 | chronic myeloproliferative disorder |
| 9962 | 3 | 20 | chronic myeloproliferative disorder |
| 8310 | 3 | 21 | clear cell adenocarcinoma |
| 8313 | 1 | 21 | clear cell adenocarcinoma |
| 8313 | 3 | 21 | clear cell adenocarcinoma |
| 9350 | 1 | 22 | craniopharyngioma |
| 9351 | 1 | 22 | craniopharyngioma |
| 8440 | 3 | 23 | cystadenocarcinoma |
| 8444 | 1 | 23 | cystadenocarcinoma |
| 8500 | 3 | 24 | ductal carcinoma |
| 8500 | 2 | 24 | ductal carcinoma |
| 8501 | 2 | 24 | ductal carcinoma |
| 8501 | 3 | 24 | ductal carcinoma |
| 8503 | 3 | 24 | ductal carcinoma |
| 8503 | 2 | 24 | ductal carcinoma |
| 8504 | 2 | 24 | ductal carcinoma |
| 8504 | 3 | 24 | ductal carcinoma |
| 8507 | 2 | 24 | ductal carcinoma |
| 8507 | 3 | 24 | ductal carcinoma |
| 8522 | 3 | 24 | ductal carcinoma |
| 8522 | 2 | 24 | ductal carcinoma |
| 8523 | 3 | 24 | ductal carcinoma |
| 8524 | 3 | 24 | ductal carcinoma |
| 8575 | 3 | 24 | ductal carcinoma |
| 9413 | 0 | 25 | dysembryoplastic neuroepithelial tumor |
| 8380 | 3 | 26 | endometroid adenocarcinoma |
| 8380 | 1 | 26 | endometroid adenocarcinoma |
| 8381 | 3 | 26 | endometroid adenocarcinoma |
| 8384 | 3 | 26 | endometroid adenocarcinoma |
| 9383 | 1 | 27 | ependymoma |
| 9391 | 3 | 27 | ependymoma |
| 9392 | 3 | 27 | ependymoma |
| 9394 | 1 | 27 | ependymoma |
| 8010 | 3 | 28 | epithelial neoplasm and carcinoma |
| 8010 | 2 | 28 | epithelial neoplasm and carcinoma |
| 8010 | 0 | 28 | epithelial neoplasm and carcinoma |
| 8012 | 3 | 28 | epithelial neoplasm and carcinoma |
| 8015 | 3 | 28 | epithelial neoplasm and carcinoma |
| 8020 | 3 | 28 | epithelial neoplasm and carcinoma |
| 8021 | 3 | 28 | epithelial neoplasm and carcinoma |
| 8022 | 3 | 28 | epithelial neoplasm and carcinoma |
| 8031 | 3 | 28 | epithelial neoplasm and carcinoma |
| 8032 | 3 | 28 | epithelial neoplasm and carcinoma |
| 8033 | 3 | 28 | epithelial neoplasm and carcinoma |
| 8046 | 3 | 28 | epithelial neoplasm and carcinoma |
| 8562 | 3 | 28 | epithelial neoplasm and carcinoma |
| 8941 | 3 | 28 | epithelial neoplasm and carcinoma |
| 8982 | 3 | 28 | epithelial neoplasm and carcinoma |
| 8810 | 3 | 29 | fibrosarcoma |
| 8811 | 3 | 29 | fibrosarcoma |
| 8814 | 3 | 29 | fibrosarcoma |
| 8815 | 3 | 29 | fibrosarcoma |
| 8815 | 0 | 29 | fibrosarcoma |
| 8821 | 1 | 29 | fibrosarcoma |
| 8830 | 3 | 29 | fibrosarcoma |
| 8832 | 3 | 29 | fibrosarcoma |
| 8840 | 3 | 29 | fibrosarcoma |
| 9260 | 3 | 30 | ewing sarcoma |
| 9000 | 3 | 31 | fibroepithelial neoplasm |
| 9000 | 1 | 31 | fibroepithelial neoplasm |
| 9020 | 3 | 31 | fibroepithelial neoplasm |
| 9020 | 1 | 31 | fibroepithelial neoplasm |
| 8330 | 3 | 32 | follicular adenocarcinoma |
| 8330 | 1 | 32 | follicular adenocarcinoma |
| 8331 | 3 | 32 | follicular adenocarcinoma |
| 8335 | 3 | 32 | follicular adenocarcinoma |
| 9590 | 3 | 33 | malignant b-cell lymphoma, non-hodgkin |
| 9591 | 3 | 33 | malignant b-cell lymphoma, non-hodgkin |
| 9596 | 3 | 33 | malignant b-cell lymphoma, non-hodgkin |
| 9670 | 3 | 33 | malignant b-cell lymphoma, non-hodgkin |
| 9671 | 3 | 33 | malignant b-cell lymphoma, non-hodgkin |
| 9673 | 3 | 33 | malignant b-cell lymphoma, non-hodgkin |
| 9675 | 3 | 33 | malignant b-cell lymphoma, non-hodgkin |
| 9679 | 3 | 33 | malignant b-cell lymphoma, non-hodgkin |
| 9680 | 3 | 33 | malignant b-cell lymphoma, non-hodgkin |
| 9684 | 3 | 33 | malignant b-cell lymphoma, non-hodgkin |
| 9689 | 3 | 33 | malignant b-cell lymphoma, non-hodgkin |
| 9690 | 3 | 33 | malignant b-cell lymphoma, non-hodgkin |
| 9691 | 3 | 33 | malignant b-cell lymphoma, non-hodgkin |
| 9695 | 3 | 33 | malignant b-cell lymphoma, non-hodgkin |
| 9698 | 3 | 33 | malignant b-cell lymphoma, non-hodgkin |
| 9699 | 3 | 33 | malignant b-cell lymphoma, non-hodgkin |
| 8936 | 1 | 34 | gastrointestinal stromal tumor |
| 8936 | 3 | 34 | gastrointestinal stromal tumor |
| 9060 | 3 | 35 | germinoma, seminoma, dysgerminoma |
| 9061 | 3 | 35 | germinoma, seminoma, dysgerminoma |
| 9062 | 3 | 35 | germinoma, seminoma, dysgerminoma |
| 9063 | 3 | 35 | germinoma, seminoma, dysgerminoma |
| 9064 | 2 | 35 | germinoma, seminoma, dysgerminoma |
| 9064 | 3 | 35 | germinoma, seminoma, dysgerminoma |
| 9073 | 1 | 35 | germinoma, seminoma, dysgerminoma |
| 9070 | 3 | 36 | embryonal carcinoma |
| 9071 | 3 | 36 | embryonal carcinoma |
| 9080 | 3 | 37 | teratoma |
| 9080 | 1 | 37 | teratoma |
| 9080 | 0 | 37 | teratoma |
| 9081 | 3 | 37 | teratoma |
| 9084 | 3 | 37 | teratoma |
| 9084 | 0 | 37 | teratoma |
| 9090 | 3 | 37 | teratoma |
| 9085 | 3 | 38 | mixed germ cell tumor |
| 9100 | 3 | 39 | choriocarcinoma |
| 9101 | 3 | 39 | choriocarcinoma |
| 9105 | 3 | 39 | choriocarcinoma |
| 9380 | 3 | 40 | glioma, glioblastoma |
| 9381 | 3 | 40 | glioma, glioblastoma |
| 9382 | 3 | 40 | glioma, glioblastoma |
| 9440 | 3 | 40 | glioma, glioblastoma |
| 9441 | 3 | 40 | glioma, glioblastoma |
| 9442 | 3 | 40 | glioma, glioblastoma |
| 9444 | 1 | 40 | glioma, glioblastoma |
| 9580 | 3 | 41 | granular cell tumor |
| 9161 | 1 | 42 | hemangioblastoma |
| 9120 | 0 | 43 | hemangioma |
| 9121 | 0 | 43 | hemangioma |
| 9122 | 0 | 43 | hemangioma |
| 9130 | 3 | 43 | hemangioma |
| 9150 | 3 | 44 | hemangiopericytoma |
| 9150 | 1 | 44 | hemangiopericytoma |
| 9150 | 0 | 44 | hemangiopericytoma |
| 8970 | 3 | 45 | hepatoblastoma |
| 8170 | 3 | 46 | hepatocellular carcinoma |
| 8171 | 3 | 46 | hepatocellular carcinoma |
| 8173 | 3 | 46 | hepatocellular carcinoma |
| 9650 | 3 | 47 | hodgkin lymphoma |
| 9651 | 3 | 47 | hodgkin lymphoma |
| 9652 | 3 | 47 | hodgkin lymphoma |
| 9653 | 3 | 47 | hodgkin lymphoma |
| 9655 | 3 | 47 | hodgkin lymphoma |
| 9659 | 3 | 47 | hodgkin lymphoma |
| 9663 | 3 | 47 | hodgkin lymphoma |
| 9665 | 3 | 47 | hodgkin lymphoma |
| 9667 | 3 | 47 | hodgkin lymphoma |
| 9140 | 3 | 48 | kaposi sarcoma |
| 8890 | 3 | 49 | leiomyoma, leiomyosarcoma |
| 8890 | 1 | 49 | leiomyoma, leiomyosarcoma |
| 9800 | 3 | 50 | leukemia |
| 8850 | 3 | 51 | liposarcoma |
| 8850 | 0 | 51 | liposarcoma |
| 8851 | 3 | 51 | liposarcoma |
| 8851 | 0 | 51 | liposarcoma |
| 8852 | 3 | 51 | liposarcoma |
| 8853 | 3 | 51 | liposarcoma |
| 8854 | 3 | 51 | liposarcoma |
| 8858 | 3 | 51 | liposarcoma |
| 8520 | 3 | 52 | lobular carcinoma |
| 8520 | 2 | 52 | lobular carcinoma |
| 9820 | 3 | 53 | lymphoid leukemia |
| 9827 | 3 | 53 | lymphoid leukemia |
| 9831 | 3 | 53 | lymphoid leukemia |
| 9750 | 3 | 54 | malignant histiocytosis |
| 9751 | 1 | 54 | malignant histiocytosis |
| 9756 | 3 | 54 | malignant histiocytosis |
| 9757 | 3 | 54 | malignant histiocytosis |
| 9701 | 3 | 55 | malignant t-cell lymphoma, non-hodgkin |
| 9702 | 3 | 55 | malignant t-cell lymphoma, non-hodgkin |
| 9705 | 3 | 55 | malignant t-cell lymphoma, non-hodgkin |
| 9708 | 3 | 55 | malignant t-cell lymphoma, non-hodgkin |
| 9709 | 3 | 55 | malignant t-cell lymphoma, non-hodgkin |
| 9714 | 3 | 55 | malignant t-cell lymphoma, non-hodgkin |
| 9717 | 3 | 55 | malignant t-cell lymphoma, non-hodgkin |
| 9718 | 3 | 55 | malignant t-cell lymphoma, non-hodgkin |
| 9719 | 3 | 55 | malignant t-cell lymphoma, non-hodgkin |
| 9741 | 3 | 56 | mast cell tumor |
| 9742 | 3 | 56 | mast cell tumor |
| 8510 | 3 | 57 | medullary carcinoma |
| 9470 | 3 | 58 | medulloblastoma |
| 8720 | 3 | 59 | melanoma |
| 8720 | 2 | 59 | melanoma |
| 8721 | 3 | 59 | melanoma |
| 8726 | 0 | 59 | melanoma |
| 8730 | 3 | 59 | melanoma |
| 8742 | 3 | 59 | melanoma |
| 8742 | 2 | 59 | melanoma |
| 8743 | 3 | 59 | melanoma |
| 8744 | 3 | 59 | melanoma |
| 8745 | 3 | 59 | melanoma |
| 9530 | 0 | 60 | meningioma |
| 9530 | 3 | 60 | meningioma |
| 9530 | 1 | 60 | meningioma |
| 9531 | 0 | 60 | meningioma |
| 9532 | 0 | 60 | meningioma |
| 9533 | 0 | 60 | meningioma |
| 9534 | 0 | 60 | meningioma |
| 9537 | 0 | 60 | meningioma |
| 9538 | 1 | 60 | meningioma |
| 9538 | 3 | 60 | meningioma |
| 9539 | 1 | 60 | meningioma |
| 8247 | 3 | 61 | merkel cell carcinoma |
| 9110 | 3 | 62 | mesonephroma |
| 9110 | 1 | 62 | mesonephroma |
| 9050 | 3 | 63 | mesothelioma |
| 9051 | 3 | 63 | mesothelioma |
| 9052 | 3 | 63 | mesothelioma |
| 9053 | 3 | 63 | mesothelioma |
| 8940 | 3 | 64 | mixed tumor |
| 8453 | 3 | 65 | mucinous and mucionus cystic tumor |
| 8470 | 3 | 65 | mucinous and mucionus cystic tumor |
| 8470 | 1 | 65 | mucinous and mucionus cystic tumor |
| 8471 | 3 | 65 | mucinous and mucionus cystic tumor |
| 8472 | 1 | 65 | mucinous and mucionus cystic tumor |
| 8473 | 1 | 65 | mucinous and mucionus cystic tumor |
| 8480 | 3 | 65 | mucinous and mucionus cystic tumor |
| 8490 | 3 | 65 | mucinous and mucionus cystic tumor |
| 8430 | 3 | 66 | mucoepidermoid neoplasms |
| 9731 | 3 | 67 | multiple myeloma |
| 9732 | 3 | 67 | multiple myeloma |
| 9733 | 3 | 67 | multiple myeloma |
| 9734 | 3 | 67 | multiple myeloma |
| 9700 | 3 | 68 | mycosis fungoides |
| 9980 | 3 | 69 | myelodysplastic syndrome |
| 9982 | 3 | 69 | myelodysplastic syndrome |
| 9983 | 3 | 69 | myelodysplastic syndrome |
| 9984 | 3 | 69 | myelodysplastic syndrome |
| 9985 | 3 | 69 | myelodysplastic syndrome |
| 9989 | 3 | 69 | myelodysplastic syndrome |
| 9860 | 3 | 70 | myeloid leukemia |
| 9930 | 3 | 70 | myeloid leukemia |
| 9961 | 3 | 71 | myelosclerosis |
| 6666 | 0 | 72 | neoplasm benign |
| 8000 | 0 | 72 | neoplasm benign |
| 8000 | 1 | 72 | neoplasm benign |
| 8000 | 3 | 73 | neoplasm malignant |
| 8003 | 3 | 73 | neoplasm malignant |
| 8960 | 3 | 74 | nephroblastoma |
| 9540 | 3 | 75 | nerve sheath tumor |
| 9560 | 3 | 75 | nerve sheath tumor |
| 9490 | 3 | 76 | neuroblastoma |
| 9500 | 3 | 76 | neuroblastoma |
| 9503 | 3 | 76 | neuroblastoma |
| 9522 | 3 | 76 | neuroblastoma |
| 8013 | 3 | 77 | neuroendocrine carcinoma |
| 8150 | 3 | 77 | neuroendocrine carcinoma |
| 8151 | 3 | 77 | neuroendocrine carcinoma |
| 8153 | 3 | 77 | neuroendocrine carcinoma |
| 8155 | 3 | 77 | neuroendocrine carcinoma |
| 8240 | 3 | 77 | neuroendocrine carcinoma |
| 8240 | 1 | 77 | neuroendocrine carcinoma |
| 8243 | 3 | 77 | neuroendocrine carcinoma |
| 8244 | 3 | 77 | neuroendocrine carcinoma |
| 8246 | 3 | 77 | neuroendocrine carcinoma |
| 8249 | 3 | 77 | neuroendocrine carcinoma |
| 8320 | 3 | 77 | neuroendocrine carcinoma |
| 8337 | 3 | 77 | neuroendocrine carcinoma |
| 9490 | 0 | 78 | neuroepitheliomatous neoplasm |
| 9492 | 0 | 78 | neuroepitheliomatous neoplasm |
| 9505 | 1 | 78 | neuroepitheliomatous neoplasm |
| 9505 | 3 | 78 | neuroepitheliomatous neoplasm |
| 9506 | 1 | 78 | neuroepitheliomatous neoplasm |
| 9508 | 3 | 78 | neuroepitheliomatous neoplasm |
| 9540 | 0 | 79 | neurofibroma |
| 9540 | 1 | 79 | neurofibroma |
| 9550 | 0 | 79 | neurofibroma |
| 9560 | 0 | 79 | neurofibroma |
| 9450 | 3 | 80 | oligodendroglioma |
| 9451 | 3 | 80 | oligodendroglioma |
| 9180 | 3 | 81 | osteosarcoma |
| 9180 | 0 | 81 | osteosarcoma |
| 9181 | 3 | 81 | osteosarcoma |
| 9183 | 3 | 81 | osteosarcoma |
| 9200 | 1 | 81 | osteosarcoma |
| 9261 | 3 | 81 | osteosarcoma |
| 8540 | 3 | 82 | paget disease |
| 8541 | 3 | 82 | paget disease |
| 8542 | 3 | 82 | paget disease |
| 8543 | 3 | 82 | paget disease |
| 8260 | 3 | 83 | papillary adenocarcinoma |
| 8050 | 2 | 84 | papillary carcinoma |
| 8050 | 3 | 84 | papillary carcinoma |
| 8051 | 3 | 84 | papillary carcinoma |
| 8052 | 3 | 84 | papillary carcinoma |
| 8340 | 3 | 84 | papillary carcinoma |
| 8341 | 3 | 84 | papillary carcinoma |
| 8343 | 3 | 84 | papillary carcinoma |
| 8680 | 3 | 85 | paraganglioma, pheochromocytoma |
| 8680 | 0 | 85 | paraganglioma, pheochromocytoma |
| 8680 | 1 | 85 | paraganglioma, pheochromocytoma |
| 8693 | 1 | 85 | paraganglioma, pheochromocytoma |
| 8700 | 3 | 85 | paraganglioma, pheochromocytoma |
| 9360 | 1 | 86 | pineoblastoma |
| 9361 | 1 | 86 | pineoblastoma |
| 9362 | 3 | 86 | pineoblastoma |
| 9950 | 3 | 87 | polycythemia vera |
| 9832 | 3 | 88 | precursor cell lymphoblastic leukemia |
| 9834 | 3 | 88 | precursor cell lymphoblastic leukemia |
| 9835 | 3 | 88 | precursor cell lymphoblastic leukemia |
| 9836 | 3 | 88 | precursor cell lymphoblastic leukemia |
| 9837 | 3 | 88 | precursor cell lymphoblastic leukemia |
| 9727 | 3 | 89 | precursor cell lymphoblastic lymphoma, non-hodgkin |
| 9728 | 3 | 89 | precursor cell lymphoblastic lymphoma, non-hodgkin |
| 9729 | 3 | 89 | precursor cell lymphoblastic lymphoma, non-hodgkin |
| 9364 | 3 | 90 | primitive and peripheral neuroectodermal tumor |
| 9365 | 3 | 90 | primitive and peripheral neuroectodermal tumor |
| 9471 | 3 | 90 | primitive and peripheral neuroectodermal tumor |
| 9473 | 3 | 90 | primitive and peripheral neuroectodermal tumor |
| 8972 | 3 | 91 | pulmonary blastoma |
| 8973 | 3 | 91 | pulmonary blastoma |
| 8312 | 3 | 92 | renal cell carcinoma |
| 8316 | 3 | 92 | renal cell carcinoma |
| 8317 | 3 | 92 | renal cell carcinoma |
| 8318 | 3 | 92 | renal cell carcinoma |
| 8319 | 3 | 92 | renal cell carcinoma |
| 8900 | 3 | 93 | rhabdomyosarcoma |
| 8901 | 3 | 93 | rhabdomyosarcoma |
| 8910 | 3 | 93 | rhabdomyosarcoma |
| 8920 | 3 | 93 | rhabdomyosarcoma |
| 8963 | 3 | 93 | rhabdomyosarcoma |
| 8800 | 3 | 94 | sarcoma or cellular fibroma |
| 8801 | 3 | 94 | sarcoma or cellular fibroma |
| 8802 | 3 | 94 | sarcoma or cellular fibroma |
| 8803 | 3 | 94 | sarcoma or cellular fibroma |
| 8804 | 3 | 94 | sarcoma or cellular fibroma |
| 8806 | 3 | 94 | sarcoma or cellular fibroma |
| 8810 | 1 | 94 | sarcoma or cellular fibroma |
| 8895 | 3 | 94 | sarcoma or cellular fibroma |
| 8933 | 3 | 94 | sarcoma or cellular fibroma |
| 8990 | 3 | 94 | sarcoma or cellular fibroma |
| 8991 | 3 | 94 | sarcoma or cellular fibroma |
| 9120 | 3 | 94 | sarcoma or cellular fibroma |
| 9133 | 3 | 94 | sarcoma or cellular fibroma |
| 9170 | 3 | 94 | sarcoma or cellular fibroma |
| 9250 | 3 | 94 | sarcoma or cellular fibroma |
| 9251 | 3 | 94 | sarcoma or cellular fibroma |
| 9581 | 3 | 94 | sarcoma or cellular fibroma |
| 8930 | 3 | 95 | stromal sarcoma |
| 8931 | 3 | 95 | stromal sarcoma |
| 8935 | 3 | 95 | stromal sarcoma |
| 8441 | 3 | 96 | serous cystic and papillary carcinoma |
| 8442 | 1 | 96 | serous cystic and papillary carcinoma |
| 8450 | 3 | 96 | serous cystic and papillary carcinoma |
| 8451 | 1 | 96 | serous cystic and papillary carcinoma |
| 8460 | 3 | 96 | serous cystic and papillary carcinoma |
| 8461 | 3 | 96 | serous cystic and papillary carcinoma |
| 8462 | 1 | 96 | serous cystic and papillary carcinoma |
| 8463 | 1 | 96 | serous cystic and papillary carcinoma |
| 8041 | 3 | 97 | small cell carcinoma |
| 8044 | 3 | 97 | small cell carcinoma |
| 8045 | 3 | 97 | small cell carcinoma |
| 8590 | 3 | 98 | specialized gonadal neoplasm |
| 8590 | 1 | 98 | specialized gonadal neoplasm |
| 8620 | 1 | 98 | specialized gonadal neoplasm |
| 8620 | 3 | 98 | specialized gonadal neoplasm |
| 8622 | 1 | 98 | specialized gonadal neoplasm |
| 8630 | 1 | 98 | specialized gonadal neoplasm |
| 8631 | 1 | 98 | specialized gonadal neoplasm |
| 8631 | 3 | 98 | specialized gonadal neoplasm |
| 8632 | 1 | 98 | specialized gonadal neoplasm |
| 8640 | 1 | 98 | specialized gonadal neoplasm |
| 8650 | 3 | 98 | specialized gonadal neoplasm |
| 8070 | 3 | 99 | squamous cell neoplasms and carcinoma |
| 8070 | 2 | 99 | squamous cell neoplasms and carcinoma |
| 8071 | 3 | 99 | squamous cell neoplasms and carcinoma |
| 8072 | 3 | 99 | squamous cell neoplasms and carcinoma |
| 8073 | 3 | 99 | squamous cell neoplasms and carcinoma |
| 8074 | 3 | 99 | squamous cell neoplasms and carcinoma |
| 8075 | 3 | 99 | squamous cell neoplasms and carcinoma |
| 8076 | 3 | 99 | squamous cell neoplasms and carcinoma |
| 8077 | 2 | 99 | squamous cell neoplasms and carcinoma |
| 8082 | 3 | 99 | squamous cell neoplasms and carcinoma |
| 8083 | 3 | 99 | squamous cell neoplasms and carcinoma |
| 8084 | 3 | 99 | squamous cell neoplasms and carcinoma |
| 9040 | 3 | 100 | synovial sarcoma |
| 9044 | 3 | 100 | synovial sarcoma |
| 8580 | 3 | 101 | thymoma |
| 8585 | 3 | 101 | thymoma |
| 8120 | 3 | 102 | transitional cell carcinoma |
| 8120 | 1 | 102 | transitional cell carcinoma |
| 8120 | 2 | 102 | transitional cell carcinoma |
| 8121 | 3 | 102 | transitional cell carcinoma |
| 8130 | 1 | 102 | transitional cell carcinoma |
| 8130 | 2 | 102 | transitional cell carcinoma |
| 8130 | 3 | 102 | transitional cell carcinoma |
| 8131 | 3 | 102 | transitional cell carcinoma |
| 9761 | 3 | 103 | waldenstrom macroglobulinemia |
| 9510 | 3 | 104 | retinoblastoma |

* All ICD-O-3 based morphologies in the Finnish Cancer Registry used for cancer diagnoses between years 1953-2008.
