# Supplementary material for: Nationwide Registry-Based Analysis of Cancer Clustering Detects Strong Familial Occurrence of Kaposi Sarcoma
Source: PLoS One. 2013 Jan 24;8(1):e55209. doi: 10.1371/journal.pone.0055209 (PMC3554690; doi:10.1371/journal.pone.0055209)
Supplement: Table S2 — ICD-O-3 based topography code groups used in the birth name-municipality based clustering. (DOCX) [file pone.0055209.s002.docx]

**Table S2. ICD-O-3 based topography code groups used in the birth name-municipality based clustering.**

| Topography (ICD-O-3)* | Topography Group | Topography Group Definition |
| --- | --- | --- |
| C00.0 | C00.0-C00.9 | Lip |
| C00.1 | C00.0-C00.9 | Lip |
| C00.2 | C00.0-C00.9 | Lip |
| C00.3 | C00.0-C00.9 | Lip |
| C00.4 | C00.0-C00.9 | Lip |
| C00.5 | C00.0-C00.9 | Lip |
| C00.6 | C00.0-C00.9 | Lip |
| C00.8 | C00.0-C00.9 | Lip |
| C00.9 | C00.0-C00.9 | Lip |
| C01.9 | C01.9-C02.9 | Tongue |
| C02.0 | C01.9-C02.9 | Tongue |
| C02.1 | C01.9-C02.9 | Tongue |
| C02.2 | C01.9-C02.9 | Tongue |
| C02.3 | C01.9-C02.9 | Tongue |
| C02.4 | C01.9-C02.9 | Tongue |
| C02.8 | C01.9-C02.9 | Tongue |
| C02.9 | C01.9-C02.9 | Tongue |
| C03.0 | C03.0-C06.9 | Mouth and gum |
| C03.1 | C03.0-C06.9 | Mouth and gum |
| C03.9 | C03.0-C06.9 | Mouth and gum |
| C04.0 | C03.0-C06.9 | Mouth and gum |
| C04.1 | C03.0-C06.9 | Mouth and gum |
| C04.8 | C03.0-C06.9 | Mouth and gum |
| C04.9 | C03.0-C06.9 | Mouth and gum |
| C05.0 | C03.0-C06.9 | Mouth and gum |
| C05.1 | C03.0-C06.9 | Mouth and gum |
| C05.2 | C03.0-C06.9 | Mouth and gum |
| C05.8 | C03.0-C06.9 | Mouth and gum |
| C05.9 | C03.0-C06.9 | Mouth and gum |
| C06.0 | C03.0-C06.9 | Mouth and gum |
| C06.1 | C03.0-C06.9 | Mouth and gum |
| C06.2 | C03.0-C06.9 | Mouth and gum |
| C06.8 | C03.0-C06.9 | Mouth and gum |
| C06.9 | C03.0-C06.9 | Mouth and gum |
| C07.9 | C07.0-C08.9 | Salivary gland |
| C08.0 | C07.0-C08.9 | Salivary gland |
| C08.1 | C07.0-C08.9 | Salivary gland |
| C08.8 | C07.0-C08.9 | Salivary gland |
| C08.9 | C07.0-C08.9 | Salivary gland |
| C09.0 | C09.0-C10.9 | Oropharynx, mesopharynx |
| C09.1 | C09.0-C10.9 | Oropharynx, mesopharynx |
| C09.8 | C09.0-C10.9 | Oropharynx, mesopharynx |
| C09.9 | C09.0-C10.9 | Oropharynx, mesopharynx |
| C10.0 | C09.0-C10.9 | Oropharynx, mesopharynx |
| C10.1 | C09.0-C10.9 | Oropharynx, mesopharynx |
| C10.2 | C09.0-C10.9 | Oropharynx, mesopharynx |
| C10.3 | C09.0-C10.9 | Oropharynx, mesopharynx |
| C10.4 | C09.0-C10.9 | Oropharynx, mesopharynx |
| C10.8 | C09.0-C10.9 | Oropharynx, mesopharynx |
| C10.9 | C09.0-C10.9 | Oropharynx, mesopharynx |
| C11.0 | C10.0-C11.9 | Nasopharynx |
| C11.1 | C10.0-C11.9 | Nasopharynx |
| C11.2 | C10.0-C11.9 | Nasopharynx |
| C11.3 | C10.0-C11.9 | Nasopharynx |
| C11.8 | C10.0-C11.9 | Nasopharynx |
| C11.9 | C10.0-C11.9 | Nasopharynx |
| C12.9 | C12.0-C13.9 | Hypopharynx |
| C13.0 | C12.0-C13.9 | Hypopharynx |
| C13.1 | C12.0-C13.9 | Hypopharynx |
| C13.2 | C12.0-C13.9 | Hypopharynx |
| C13.8 | C12.0-C13.9 | Hypopharynx |
| C13.9 | C12.0-C13.9 | Hypopharynx |
| C14.0 | C14.0-C14.8 | Pharynx |
| C14.2 | C14.0-C14.8 | Pharynx |
| C14.8 | C14.0-C14.8 | Pharynx |
| C15.0 | C15.0-C15.9 | Esophagus |
| C15.1 | C15.0-C15.9 | Esophagus |
| C15.2 | C15.0-C15.9 | Esophagus |
| C15.3 | C15.0-C15.9 | Esophagus |
| C15.4 | C15.0-C15.9 | Esophagus |
| C15.5 | C15.0-C15.9 | Esophagus |
| C15.8 | C15.0-C15.9 | Esophagus |
| C15.9 | C15.0-C15.9 | Esophagus |
| C16.0 | C16.0-C16.9 | Stomach |
| C16.1 | C16.0-C16.9 | Stomach |
| C16.2 | C16.0-C16.9 | Stomach |
| C16.3 | C16.0-C16.9 | Stomach |
| C16.4 | C16.0-C16.9 | Stomach |
| C16.5 | C16.0-C16.9 | Stomach |
| C16.6 | C16.0-C16.9 | Stomach |
| C16.8 | C16.0-C16.9 | Stomach |
| C16.9 | C16.0-C16.9 | Stomach |
| C17.0 | C17.0-C17.9 | Small intestine |
| C17.1 | C17.0-C17.9 | Small intestine |
| C17.2 | C17.0-C17.9 | Small intestine |
| C17.3 | C17.0-C17.9 | Small intestine |
| C17.8 | C17.0-C17.9 | Small intestine |
| C17.9 | C17.0-C17.9 | Small intestine |
| C18.0 | C18.0-C18.9,C26.0 | Large intestine |
| C18.1 | C18.0-C18.9,C26.0 | Large intestine |
| C18.2 | C18.0-C18.9,C26.0 | Large intestine |
| C18.3 | C18.0-C18.9,C26.0 | Large intestine |
| C18.4 | C18.0-C18.9,C26.0 | Large intestine |
| C18.5 | C18.0-C18.9,C26.0 | Large intestine |
| C18.6 | C18.0-C18.9,C26.0 | Large intestine |
| C18.7 | C18.0-C18.9,C26.0 | Large intestine |
| C18.8 | C18.0-C18.9,C26.0 | Large intestine |
| C18.9 | C18.0-C18.9,C26.0 | Large intestine |
| C26.0 | C18.0-C18.9,C26.0 | Large intestine |
| C19.9 | C19.0-C21.9 | Rectum |
| C20.9 | C19.0-C21.9 | Rectum |
| C21.0 | C19.0-C21.9 | Rectum |
| C21.1 | C19.0-C21.9 | Rectum |
| C21.2 | C19.0-C21.9 | Rectum |
| C21.8 | C19.0-C21.9 | Rectum |
| C22.0 | C22.0-C22.1 | Hepar |
| C22.1 | C22.0-C22.1 | Hepar |
| C23.9 | C23.9-C24.9 | Gallbladder and bile duct |
| C24.0 | C23.9-C24.9 | Gallbladder and bile duct |
| C24.1 | C23.9-C24.9 | Gallbladder and bile duct |
| C24.8 | C23.9-C24.9 | Gallbladder and bile duct |
| C24.9 | C23.9-C24.9 | Gallbladder and bile duct |
| C25.0 | C25.0-C25.9 | Pancreas |
| C25.1 | C25.0-C25.9 | Pancreas |
| C25.2 | C25.0-C25.9 | Pancreas |
| C25.3 | C25.0-C25.9 | Pancreas |
| C25.4 | C25.0-C25.9 | Pancreas |
| C25.7 | C25.0-C25.9 | Pancreas |
| C25.8 | C25.0-C25.9 | Pancreas |
| C25.9 | C25.0-C25.9 | Pancreas |
| C26.8 | C26.8-C26.9 | Digestive system, other unspecified |
| C26.9 | C26.8-C26.9 | Digestive system, other unspecified |
| C30.0 | C30.0-C31.9 | Nose |
| C30.1 | C30.0-C31.9 | Nose |
| C31.0 | C30.0-C31.9 | Nose |
| C31.1 | C30.0-C31.9 | Nose |
| C31.2 | C30.0-C31.9 | Nose |
| C31.3 | C30.0-C31.9 | Nose |
| C31.8 | C30.0-C31.9 | Nose |
| C31.9 | C30.0-C31.9 | Nose |
| C32.0 | C32.0-C32.9 | Larynx |
| C32.1 | C32.0-C32.9 | Larynx |
| C32.2 | C32.0-C32.9 | Larynx |
| C32.3 | C32.0-C32.9 | Larynx |
| C32.8 | C32.0-C32.9 | Larynx |
| C32.9 | C32.0-C32.9 | Larynx |
| C33.9 | C34.0-C34.9 | Lung and trachea |
| C34.0 | C34.0-C34.9 | Lung and trachea |
| C34.1 | C34.0-C34.9 | Lung and trachea |
| C34.2 | C34.0-C34.9 | Lung and trachea |
| C34.3 | C34.0-C34.9 | Lung and trachea |
| C34.8 | C34.0-C34.9 | Lung and trachea |
| C34.9 | C34.0-C34.9 | Lung and trachea |
| C37.9 | C37.0-C37.9 | Thymus |
| C38.0 | C38.0-C38.9 | Hearth |
| C38.1 | C38.1-C38.3,C38.8 | Mediastinum |
| C38.2 | C38.1-C38.3,C38.8 | Mediastinum |
| C38.3 | C38.1-C38.3,C38.8 | Mediastinum |
| C38.4 | C38.4 | Mesothelium |
| C38.8 | C38.1-C38.3,C38.8 | Mediastinum |
| C39.0 | C39.0-C39.9 | Respiratory tract or thoracic cavity, other unspecified location |
| C39.8 | C39.0-C39.9 | Respiratory tract or thoracic cavity, other unspecified location |
| C39.9 | C39.0-C39.9 | Respiratory tract or thoracic cavity, other unspecified location |
| C40.0 | C40.0-C41.9 | Bone |
| C40.1 | C40.0-C41.9 | Bone |
| C40.2 | C40.0-C41.9 | Bone |
| C40.3 | C40.0-C41.9 | Bone |
| C40.8 | C40.0-C41.9 | Bone |
| C40.9 | C40.0-C41.9 | Bone |
| C41.0 | C40.0-C41.9 | Bone |
| C41.1 | C40.0-C41.9 | Bone |
| C41.2 | C40.0-C41.9 | Bone |
| C41.3 | C40.0-C41.9 | Bone |
| C41.4 | C40.0-C41.9 | Bone |
| C41.8 | C40.0-C41.9 | Bone |
| C41.9 | C40.0-C41.9 | Bone |
| C42.0 | C42.0-C42.4 | Hematopoetic or reticuloendothelial system |
| C42.1 | C42.0-C42.4 | Hematopoetic or reticuloendothelial system |
| C42.2 | C42.0-C42.4 | Hematopoetic or reticuloendothelial system |
| C42.3 | C42.0-C42.4 | Hematopoetic or reticuloendothelial system |
| C42.4 | C42.0-C42.4 | Hematopoetic or reticuloendothelial system |
| C44.0 | C44.0-C44.9 | Skin |
| C44.1 | C44.0-C44.9 | Skin |
| C44.2 | C44.0-C44.9 | Skin |
| C44.3 | C44.0-C44.9 | Skin |
| C44.4 | C44.0-C44.9 | Skin |
| C44.5 | C44.0-C44.9 | Skin |
| C44.6 | C44.0-C44.9 | Skin |
| C44.7 | C44.0-C44.9 | Skin |
| C44.8 | C44.0-C44.9 | Skin |
| C44.9 | C44.0-C44.9 | Skin |
| C47.0 | C47.0-C47.9 | Peripheral nervous system |
| C47.1 | C47.0-C47.9 | Peripheral nervous system |
| C47.2 | C47.0-C47.9 | Peripheral nervous system |
| C47.3 | C47.0-C47.9 | Peripheral nervous system |
| C47.4 | C47.0-C47.9 | Peripheral nervous system |
| C47.5 | C47.0-C47.9 | Peripheral nervous system |
| C47.6 | C47.0-C47.9 | Peripheral nervous system |
| C47.8 | C47.0-C47.9 | Peripheral nervous system |
| C47.9 | C47.0-C47.9 | Peripheral nervous system |
| C48.0 | C48.0-C48.8 | Peritoneum |
| C48.1 | C48.0-C48.8 | Peritoneum |
| C48.2 | C48.0-C48.8 | Peritoneum |
| C48.8 | C48.0-C48.8 | Peritoneum |
| C49.0 | C49.0-C49.9 | Connective tissue |
| C49.1 | C49.0-C49.9 | Connective tissue |
| C49.2 | C49.0-C49.9 | Connective tissue |
| C49.3 | C49.0-C49.9 | Connective tissue |
| C49.4 | C49.0-C49.9 | Connective tissue |
| C49.5 | C49.0-C49.9 | Connective tissue |
| C49.6 | C49.0-C49.9 | Connective tissue |
| C49.8 | C49.0-C49.9 | Connective tissue |
| C49.9 | C49.0-C49.9 | Connective tissue |
| C50.0 | C50.0-C50.9 | Breast |
| C50.1 | C50.0-C50.9 | Breast |
| C50.2 | C50.0-C50.9 | Breast |
| C50.3 | C50.0-C50.9 | Breast |
| C50.4 | C50.0-C50.9 | Breast |
| C50.5 | C50.0-C50.9 | Breast |
| C50.6 | C50.0-C50.9 | Breast |
| C50.8 | C50.0-C50.9 | Breast |
| C50.9 | C50.0-C50.9 | Breast |
| C51.0 | C51.0-C52.9 | Vulva and vagina |
| C51.1 | C51.0-C52.9 | Vulva and vagina |
| C51.2 | C51.0-C52.9 | Vulva and vagina |
| C51.8 | C51.0-C52.9 | Vulva and vagina |
| C51.9 | C51.0-C52.9 | Vulva and vagina |
| C52.9 | C51.0-C52.9 | Vulva and vagina |
| C53.0 | C53.0-C53.8 | Endo- and Exocervix |
| C53.1 | C53.0-C53.8 | Endo- and Exocervix |
| C53.8 | C53.0-C53.8 | Endo- and Exocervix |
| C53.9 | C53.9-C54.9 | Corpus uteri and Cervix |
| C54.0 | C53.9-C54.9 | Corpus uteri and Cervix |
| C54.1 | C53.9-C54.9 | Corpus uteri and Cervix |
| C54.2 | C53.9-C54.9 | Corpus uteri and Cervix |
| C54.3 | C53.9-C54.9 | Corpus uteri and Cervix |
| C54.8 | C53.9-C54.9 | Corpus uteri and Cervix |
| C54.9 | C53.9-C54.9 | Corpus uteri and Cervix |
| C55.9 | C55.0-C55.9 | Uterus |
| C56.9 | C56.0-C56.9 | Ovary |
| C57.0 | C57.0-C57.9 | Female genitals, other unspecified location |
| C57.1 | C57.0-C57.9 | Female genitals, other unspecified location |
| C57.2 | C57.0-C57.9 | Female genitals, other unspecified location |
| C57.3 | C57.0-C57.9 | Female genitals, other unspecified location |
| C57.4 | C57.0-C57.9 | Female genitals, other unspecified location |
| C57.7 | C57.0-C57.9 | Female genitals, other unspecified location |
| C57.8 | C57.0-C57.9 | Female genitals, other unspecified location |
| C57.9 | C57.0-C57.9 | Female genitals, other unspecified location |
| C58.9 | C58.0-C58.9 | Placenta |
| C60.0 | C60.0-C60.9 | Penis |
| C60.1 | C60.0-C60.9 | Penis |
| C60.2 | C60.0-C60.9 | Penis |
| C60.8 | C60.0-C60.9 | Penis |
| C60.9 | C60.0-C60.9 | Penis |
| C61.9 | C61.0-C69.0 | Prostate |
| C62.0 | C62.0-C62.9 | Testis |
| C62.1 | C62.0-C62.9 | Testis |
| C62.9 | C62.0-C62.9 | Testis |
| C63.0 | C63.0-C63.9 | Male genitals, other uspecified location |
| C63.1 | C63.0-C63.9 | Male genitals, other uspecified location |
| C63.2 | C63.0-C63.9 | Male genitals, other uspecified location |
| C63.7 | C63.0-C63.9 | Male genitals, other uspecified location |
| C63.8 | C63.0-C63.9 | Male genitals, other uspecified location |
| C63.9 | C63.0-C63.9 | Male genitals, other uspecified location |
| C64.9 | C64.0-C64.9 | Kidney |
| C65.9 | C64.0-C64.9 | Kidney |
| C66.9 | C66.0-C68.9 | Urinary bladder and ureter |
| C67.0 | C66.0-C68.9 | Urinary bladder and ureter |
| C67.1 | C66.0-C68.9 | Urinary bladder and ureter |
| C67.2 | C66.0-C68.9 | Urinary bladder and ureter |
| C67.3 | C66.0-C68.9 | Urinary bladder and ureter |
| C67.4 | C66.0-C68.9 | Urinary bladder and ureter |
| C67.5 | C66.0-C68.9 | Urinary bladder and ureter |
| C67.6 | C66.0-C68.9 | Urinary bladder and ureter |
| C67.7 | C66.0-C68.9 | Urinary bladder and ureter |
| C67.8 | C66.0-C68.9 | Urinary bladder and ureter |
| C67.9 | C66.0-C68.9 | Urinary bladder and ureter |
| C68.0 | C66.0-C68.9 | Urinary bladder and ureter |
| C68.1 | C66.0-C68.9 | Urinary bladder and ureter |
| C68.8 | C66.0-C68.9 | Urinary bladder and ureter |
| C68.9 | C66.0-C68.9 | Urinary bladder and ureter |
| C69.0 | C69.0-C69.9 | Eye |
| C69.1 | C69.0-C69.9 | Eye |
| C69.2 | C69.0-C69.9 | Eye |
| C69.3 | C69.0-C69.9 | Eye |
| C69.4 | C69.0-C69.9 | Eye |
| C69.5 | C69.0-C69.9 | Eye |
| C69.6 | C69.0-C69.9 | Eye |
| C69.8 | C69.0-C69.9 | Eye |
| C69.9 | C69.0-C69.9 | Eye |
| C70.0 | C70.0-C72.9,C75.2 | Central nervous system |
| C70.1 | C70.0-C72.9,C75.2 | Central nervous system |
| C70.9 | C70.0-C72.9,C75.2 | Central nervous system |
| C71.0 | C70.0-C72.9,C75.2 | Central nervous system |
| C71.1 | C70.0-C72.9,C75.2 | Central nervous system |
| C71.2 | C70.0-C72.9,C75.2 | Central nervous system |
| C71.3 | C70.0-C72.9,C75.2 | Central nervous system |
| C71.4 | C70.0-C72.9,C75.2 | Central nervous system |
| C71.5 | C70.0-C72.9,C75.2 | Central nervous system |
| C71.6 | C70.0-C72.9,C75.2 | Central nervous system |
| C71.7 | C70.0-C72.9,C75.2 | Central nervous system |
| C71.8 | C70.0-C72.9,C75.2 | Central nervous system |
| C71.9 | C70.0-C72.9,C75.2 | Central nervous system |
| C72.0 | C70.0-C72.9,C75.2 | Central nervous system |
| C72.1 | C70.0-C72.9,C75.2 | Central nervous system |
| C72.2 | C70.0-C72.9,C75.2 | Central nervous system |
| C72.3 | C70.0-C72.9,C75.2 | Central nervous system |
| C72.4 | C70.0-C72.9,C75.2 | Central nervous system |
| C72.5 | C70.0-C72.9,C75.2 | Central nervous system |
| C72.8 | C70.0-C72.9,C75.2 | Central nervous system |
| C72.9 | C70.0-C72.9,C75.2 | Central nervous system |
| C75.2 | C70.0-C72.9,C75.2 | Central nervous system |
| C73.9 | C73.9 | Thyroid gland |
| C74.0 | C74.0-C74.9 | Adrenal gland |
| C74.1 | C74.0-C74.9 | Adrenal gland |
| C74.9 | C74.0-C74.9 | Adrenal gland |
| C75.0 | C75.0 | Parathyroid gland |
| C75.1 | C75.1 | Pituitary gland |
| C75.3 | C75.3 | Pineal gland |
| C75.4 | C75.4-C75.9 | Endocrineal gland, other unspecified location |
| C75.5 | C75.4-C75.9 | Endocrineal gland, other unspecified location |
| C75.8 | C75.4-C75.9 | Endocrineal gland, other unspecified location |
| C75.9 | C75.4-C75.9 | Endocrineal gland, other unspecified location |
| C76.0 | C76.0-C76.9,C80.9 | Unknown, other or unspecified location |
| C76.1 | C76.0-C76.9,C80.9 | Unknown, other or unspecified location |
| C76.2 | C76.0-C76.9,C80.9 | Unknown, other or unspecified location |
| C76.3 | C76.0-C76.9,C80.9 | Unknown, other or unspecified location |
| C76.4 | C76.0-C76.9,C80.9 | Unknown, other or unspecified location |
| C76.5 | C76.0-C76.9,C80.9 | Unknown, other or unspecified location |
| C76.7 | C76.0-C76.9,C80.9 | Unknown, other or unspecified location |
| C76.8 | C76.0-C76.9,C80.9 | Unknown, other or unspecified location |
| C80.9 | C76.0-C76.9,C80.9 | Unknown, other or unspecified location |
| C77.0 | C77.0-C77.9 | Lymph node (Hodgkin and Non-Hodgkin) |
| C77.1 | C77.0-C77.9 | Lymph node (Hodgkin and Non-Hodgkin) |
| C77.2 | C77.0-C77.9 | Lymph node (Hodgkin and Non-Hodgkin) |
| C77.3 | C77.0-C77.9 | Lymph node (Hodgkin and Non-Hodgkin) |
| C77.4 | C77.0-C77.9 | Lymph node (Hodgkin and Non-Hodgkin) |
| C77.5 | C77.0-C77.9 | Lymph node (Hodgkin and Non-Hodgkin) |
| C77.8 | C77.0-C77.9 | Lymph node (Hodgkin and Non-Hodgkin) |
| C77.9 | C77.0-C77.9 | Lymph node (Hodgkin and Non-Hodgkin) |

* All ICD-O-3 based topographies in the Finnish Cancer Registry used for cancer diagnoses between years 1953-2008.
