# Supplementary material for: Nationwide Registry-Based Analysis of Cancer Clustering Detects Strong Familial Occurrence of Kaposi Sarcoma
Source: PLoS One. 2013 Jan 24;8(1):e55209. doi: 10.1371/journal.pone.0055209 (PMC3554690; doi:10.1371/journal.pone.0055209)
Supplement: Table S3 — Topography and morphology combinations in the birth name-municipality based clustering, distribution of patients, and cluster scores. (DOCX) [file pone.0055209.s003.docx]

**Table S3. Topography and morphology combinations in the birth name-municipality based clustering, distribution of patients, and cluster scores.**

| Topography Group | Morphology Group | Number of patients in FCR | Number of patients in  MN-clusters  (CI low≥1) | Number of patients in  MN-clusters (CI low≥10) | Cluster score | p-value  (two-sided, FDR adjusted) |
| --- | --- | --- | --- | --- | --- | --- |
| C00.0-C00.9 | 1 | 2 | 0 | 0 | 0 | NA |
| C00.0-C00.9 | 4 | 14 | 0 | 0 | 0 | NA |
| C00.0-C00.9 | 5 | 18 | 0 | 0 | 0 | NA |
| C00.0-C00.9 | 28 | 133 | 0 | 0 | 0 | NA |
| C00.0-C00.9 | 33 | 6 | 0 | 0 | 0 | NA |
| C00.0-C00.9 | 59 | 4 | 0 | 0 | 0 | NA |
| C00.0-C00.9 | 61 | 2 | 0 | 0 | 0 | NA |
| C00.0-C00.9 | 66 | 7 | 0 | 0 | 0 | NA |
| C00.0-C00.9 | 73 | 77 | 0 | 0 | 0 | NA |
| C00.0-C00.9 | 84 | 9 | 0 | 0 | 0 | NA |
| C00.0-C00.9 | 99 | 7167 | 728 | 46 | 0.35 | 1.04E-05 |
| C01.9-C02.9 | 4 | 16 | 0 | 0 | 0 | NA |
| C01.9-C02.9 | 5 | 34 | 0 | 0 | 0 | NA |
| C01.9-C02.9 | 28 | 54 | 0 | 0 | 0 | NA |
| C01.9-C02.9 | 29 | 5 | 0 | 0 | 0 | NA |
| C01.9-C02.9 | 33 | 70 | 0 | 0 | 0 | NA |
| C01.9-C02.9 | 48 | 2 | 0 | 0 | 0 | NA |
| C01.9-C02.9 | 66 | 3 | 0 | 0 | 0 | NA |
| C01.9-C02.9 | 67 | 2 | 0 | 0 | 0 | NA |
| C01.9-C02.9 | 73 | 46 | 0 | 0 | 0 | NA |
| C01.9-C02.9 | 84 | 7 | 0 | 0 | 0 | NA |
| C01.9-C02.9 | 93 | 3 | 0 | 0 | 0 | NA |
| C01.9-C02.9 | 94 | 4 | 0 | 0 | 0 | NA |
| C01.9-C02.9 | 99 | 2854 | 107 | 16 | 0.3 | 3.19E-02 |
| C03.0-C06.9 | 1 | 12 | 0 | 0 | 0 | NA |
| C03.0-C06.9 | 4 | 90 | 0 | 0 | 0 | NA |
| C03.0-C06.9 | 5 | 134 | 0 | 0 | 0 | NA |
| C03.0-C06.9 | 6 | 2 | 0 | 0 | 0 | NA |
| C03.0-C06.9 | 10 | 2 | 0 | 0 | 0 | NA |
| C03.0-C06.9 | 21 | 3 | 0 | 0 | 0 | NA |
| C03.0-C06.9 | 28 | 61 | 0 | 0 | 0 | NA |
| C03.0-C06.9 | 29 | 8 | 0 | 0 | 0 | NA |
| C03.0-C06.9 | 33 | 155 | 0 | 0 | 0 | NA |
| C03.0-C06.9 | 48 | 2 | 0 | 0 | 0 | NA |
| C03.0-C06.9 | 49 | 2 | 0 | 0 | 0 | NA |
| C03.0-C06.9 | 59 | 35 | 0 | 0 | 0 | NA |
| C03.0-C06.9 | 64 | 5 | 0 | 0 | 0 | NA |
| C03.0-C06.9 | 65 | 4 | 0 | 0 | 0 | NA |
| C03.0-C06.9 | 66 | 9 | 0 | 0 | 0 | NA |
| C03.0-C06.9 | 67 | 3 | 0 | 0 | 0 | NA |
| C03.0-C06.9 | 73 | 52 | 0 | 0 | 0 | NA |
| C03.0-C06.9 | 84 | 13 | 0 | 0 | 0 | NA |
| C03.0-C06.9 | 93 | 4 | 0 | 0 | 0 | NA |
| C03.0-C06.9 | 94 | 15 | 0 | 0 | 0 | NA |
| C03.0-C06.9 | 99 | 2579 | 99 | 16 | 0.34 | 1.38E-02 |
| C07.0-C08.9 | 1 | 190 | 0 | 0 | 0 | NA |
| C07.0-C08.9 | 4 | 331 | 0 | 0 | 0 | NA |
| C07.0-C08.9 | 5 | 421 | 4 | 0 | 0 | NA |
| C07.0-C08.9 | 14 | 5 | 0 | 0 | 0 | NA |
| C07.0-C08.9 | 16 | 2 | 0 | 0 | 0 | NA |
| C07.0-C08.9 | 23 | 8 | 0 | 0 | 0 | NA |
| C07.0-C08.9 | 24 | 63 | 0 | 0 | 0 | NA |
| C07.0-C08.9 | 28 | 240 | 0 | 0 | 0 | NA |
| C07.0-C08.9 | 29 | 5 | 0 | 0 | 0 | NA |
| C07.0-C08.9 | 33 | 302 | 4 | 0 | 0 | NA |
| C07.0-C08.9 | 47 | 6 | 0 | 0 | 0 | NA |
| C07.0-C08.9 | 59 | 5 | 0 | 0 | 0 | NA |
| C07.0-C08.9 | 61 | 2 | 0 | 0 | 0 | NA |
| C07.0-C08.9 | 64 | 128 | 0 | 0 | 0 | NA |
| C07.0-C08.9 | 65 | 34 | 0 | 0 | 0 | NA |
| C07.0-C08.9 | 66 | 30 | 0 | 0 | 0 | NA |
| C07.0-C08.9 | 73 | 129 | 0 | 0 | 0 | NA |
| C07.0-C08.9 | 83 | 14 | 0 | 0 | 0 | NA |
| C07.0-C08.9 | 94 | 4 | 0 | 0 | 0 | NA |
| C07.0-C08.9 | 97 | 4 | 0 | 0 | 0 | NA |
| C07.0-C08.9 | 99 | 431 | 6 | 2 | 0.25 | 4.36E-01 |
| C07.0-C08.9 | 100 | 3 | 0 | 0 | 0 | NA |
| C09.0-C10.9 | 4 | 6 | 0 | 0 | 0 | NA |
| C09.0-C10.9 | 5 | 6 | 0 | 0 | 0 | NA |
| C09.0-C10.9 | 13 | 3 | 0 | 0 | 0 | NA |
| C09.0-C10.9 | 28 | 59 | 0 | 0 | 0 | NA |
| C09.0-C10.9 | 33 | 912 | 10 | 0 | 0 | NA |
| C09.0-C10.9 | 47 | 3 | 0 | 0 | 0 | NA |
| C09.0-C10.9 | 55 | 6 | 0 | 0 | 0 | NA |
| C09.0-C10.9 | 67 | 6 | 0 | 0 | 0 | NA |
| C09.0-C10.9 | 73 | 34 | 0 | 0 | 0 | NA |
| C09.0-C10.9 | 94 | 5 | 0 | 0 | 0 | NA |
| C09.0-C10.9 | 99 | 1066 | 29 | 8 | 0.41 | 3.24E-02 |
| C09.0-C10.9 | 102 | 3 | 0 | 0 | 0 | NA |
| C10.0-C11.9 | 4 | 11 | 0 | 0 | 0 | NA |
| C10.0-C11.9 | 5 | 14 | 0 | 0 | 0 | NA |
| C10.0-C11.9 | 13 | 2 | 0 | 0 | 0 | NA |
| C10.0-C11.9 | 17 | 3 | 0 | 0 | 0 | NA |
| C10.0-C11.9 | 28 | 117 | 0 | 0 | 0 | NA |
| C10.0-C11.9 | 33 | 151 | 0 | 0 | 0 | NA |
| C10.0-C11.9 | 47 | 3 | 0 | 0 | 0 | NA |
| C10.0-C11.9 | 59 | 4 | 0 | 0 | 0 | NA |
| C10.0-C11.9 | 65 | 4 | 0 | 0 | 0 | NA |
| C10.0-C11.9 | 67 | 2 | 0 | 0 | 0 | NA |
| C10.0-C11.9 | 73 | 33 | 0 | 0 | 0 | NA |
| C10.0-C11.9 | 93 | 2 | 0 | 0 | 0 | NA |
| C10.0-C11.9 | 94 | 4 | 0 | 0 | 0 | NA |
| C10.0-C11.9 | 99 | 430 | 6 | 6 | 0.75 | 5.68E-03 |
| C10.0-C11.9 | 102 | 24 | 0 | 0 | 0 | NA |
| C12.0-C13.9 | 4 | 3 | 0 | 0 | 0 | NA |
| C12.0-C13.9 | 28 | 45 | 0 | 0 | 0 | NA |
| C12.0-C13.9 | 33 | 15 | 0 | 0 | 0 | NA |
| C12.0-C13.9 | 49 | 2 | 0 | 0 | 0 | NA |
| C12.0-C13.9 | 73 | 36 | 0 | 0 | 0 | NA |
| C12.0-C13.9 | 99 | 887 | 17 | 11 | 0.67 | 3.71E-04 |
| C14.0-C14.8 | 28 | 10 | 0 | 0 | 0 | NA |
| C14.0-C14.8 | 33 | 18 | 0 | 0 | 0 | NA |
| C14.0-C14.8 | 73 | 12 | 0 | 0 | 0 | NA |
| C14.0-C14.8 | 99 | 91 | 0 | 0 | 0 | NA |
| C15.0-C15.9 | 4 | 1447 | 22 | 4 | 0.15 | 1.00E+00 |
| C15.0-C15.9 | 5 | 9 | 0 | 0 | 0 | NA |
| C15.0-C15.9 | 6 | 5 | 0 | 0 | 0 | NA |
| C15.0-C15.9 | 14 | 2 | 0 | 0 | 0 | NA |
| C15.0-C15.9 | 28 | 820 | 24 | 10 | 0.66 | 7.73E-04 |
| C15.0-C15.9 | 33 | 23 | 0 | 0 | 0 | NA |
| C15.0-C15.9 | 49 | 8 | 0 | 0 | 0 | NA |
| C15.0-C15.9 | 59 | 7 | 0 | 0 | 0 | NA |
| C15.0-C15.9 | 65 | 54 | 0 | 0 | 0 | NA |
| C15.0-C15.9 | 73 | 1078 | 37 | 12 | 0.6 | 4.77E-04 |
| C15.0-C15.9 | 77 | 15 | 0 | 0 | 0 | NA |
| C15.0-C15.9 | 83 | 2 | 0 | 0 | 0 | NA |
| C15.0-C15.9 | 94 | 5 | 0 | 0 | 0 | NA |
| C15.0-C15.9 | 97 | 19 | 0 | 0 | 0 | NA |
| C15.0-C15.9 | 99 | 5514 | 308 | 46 | 0.45 | 8.37E-09 |
| C16.0-C16.9 | 4 | 27371 | 2532 | 32 | 0.06 | 3.08E-08 |
| C16.0-C16.9 | 6 | 3 | 0 | 0 | 0 | NA |
| C16.0-C16.9 | 28 | 4727 | 223 | 20 | 0.23 | 1.66E-01 |
| C16.0-C16.9 | 29 | 8 | 0 | 0 | 0 | NA |
| C16.0-C16.9 | 33 | 1838 | 66 | 12 | 0.35 | 3.19E-02 |
| C16.0-C16.9 | 34 | 336 | 2 | 2 | 0.32 | 3.36E-01 |
| C16.0-C16.9 | 49 | 174 | 2 | 2 | 0.62 | 1.55E-01 |
| C16.0-C16.9 | 55 | 4 | 0 | 0 | 0 | NA |
| C16.0-C16.9 | 59 | 2 | 0 | 0 | 0 | NA |
| C16.0-C16.9 | 65 | 3241 | 146 | 24 | 0.4 | 2.30E-04 |
| C16.0-C16.9 | 67 | 4 | 0 | 0 | 0 | NA |
| C16.0-C16.9 | 73 | 5045 | 228 | 14 | 0.15 | 1.00E+00 |
| C16.0-C16.9 | 77 | 689 | 14 | 6 | 0.47 | 3.72E-02 |
| C16.0-C16.9 | 83 | 152 | 0 | 0 | 0 | NA |
| C16.0-C16.9 | 94 | 43 | 0 | 0 | 0 | NA |
| C16.0-C16.9 | 97 | 6 | 0 | 0 | 0 | NA |
| C16.0-C16.9 | 99 | 127 | 0 | 0 | 0 | NA |
| C17.0-C17.9 | 4 | 787 | 14 | 6 | 0.41 | 5.77E-02 |
| C17.0-C17.9 | 13 | 2 | 0 | 0 | 0 | NA |
| C17.0-C17.9 | 28 | 92 | 0 | 0 | 0 | NA |
| C17.0-C17.9 | 29 | 12 | 0 | 0 | 0 | NA |
| C17.0-C17.9 | 30 | 2 | 0 | 0 | 0 | NA |
| C17.0-C17.9 | 33 | 700 | 8 | 6 | 0.46 | 3.88E-02 |
| C17.0-C17.9 | 34 | 179 | 0 | 0 | 0 | NA |
| C17.0-C17.9 | 49 | 179 | 2 | 2 | 0.6 | 1.59E-01 |
| C17.0-C17.9 | 51 | 3 | 0 | 0 | 0 | NA |
| C17.0-C17.9 | 55 | 33 | 0 | 0 | 0 | NA |
| C17.0-C17.9 | 59 | 5 | 0 | 0 | 0 | NA |
| C17.0-C17.9 | 65 | 30 | 0 | 0 | 0 | NA |
| C17.0-C17.9 | 73 | 156 | 0 | 0 | 0 | NA |
| C17.0-C17.9 | 77 | 1154 | 26 | 21 | 0.98 | 1.19E-09 |
| C17.0-C17.9 | 83 | 7 | 0 | 0 | 0 | NA |
| C17.0-C17.9 | 94 | 38 | 0 | 0 | 0 | NA |
| C17.0-C17.9 | 97 | 3 | 0 | 0 | 0 | NA |
| C18.0-C18.9,C26.0 | 4 | 33091 | 2898 | 50 | 0.08 | 3.26E-06 |
| C18.0-C18.9,C26.0 | 6 | 4 | 0 | 0 | 0 | NA |
| C18.0-C18.9,C26.0 | 13 | 5 | 0 | 0 | 0 | NA |
| C18.0-C18.9,C26.0 | 14 | 3 | 0 | 0 | 0 | NA |
| C18.0-C18.9,C26.0 | 23 | 5 | 0 | 0 | 0 | NA |
| C18.0-C18.9,C26.0 | 28 | 1276 | 23 | 7 | 0.3 | 1.67E-01 |
| C18.0-C18.9,C26.0 | 29 | 4 | 0 | 0 | 0 | NA |
| C18.0-C18.9,C26.0 | 33 | 310 | 2 | 2 | 0.35 | 3.05E-01 |
| C18.0-C18.9,C26.0 | 34 | 24 | 0 | 0 | 0 | NA |
| C18.0-C18.9,C26.0 | 49 | 45 | 0 | 0 | 0 | NA |
| C18.0-C18.9,C26.0 | 51 | 7 | 0 | 0 | 0 | NA |
| C18.0-C18.9,C26.0 | 55 | 4 | 0 | 0 | 0 | NA |
| C18.0-C18.9,C26.0 | 57 | 3 | 0 | 0 | 0 | NA |
| C18.0-C18.9,C26.0 | 65 | 2935 | 106 | 20 | 0.37 | 2.69E-03 |
| C18.0-C18.9,C26.0 | 67 | 2 | 0 | 0 | 0 | NA |
| C18.0-C18.9,C26.0 | 73 | 3112 | 121 | 22 | 0.38 | 7.67E-04 |
| C18.0-C18.9,C26.0 | 77 | 1950 | 51 | 13 | 0.36 | 1.47E-02 |
| C18.0-C18.9,C26.0 | 83 | 391 | 4 | 4 | 0.55 | 5.70E-02 |
| C18.0-C18.9,C26.0 | 84 | 3 | 0 | 0 | 0 | NA |
| C18.0-C18.9,C26.0 | 94 | 15 | 0 | 0 | 0 | NA |
| C18.0-C18.9,C26.0 | 97 | 3 | 0 | 0 | 0 | NA |
| C18.0-C18.9,C26.0 | 99 | 14 | 0 | 0 | 0 | NA |
| C19.0-C21.9 | 4 | 25051 | 1921 | 32 | 0.07 | 1.39E-06 |
| C19.0-C21.9 | 5 | 5 | 0 | 0 | 0 | NA |
| C19.0-C21.9 | 11 | 49 | 0 | 0 | 0 | NA |
| C19.0-C21.9 | 28 | 719 | 6 | 2 | 0.15 | 1.00E+00 |
| C19.0-C21.9 | 33 | 58 | 0 | 0 | 0 | NA |
| C19.0-C21.9 | 34 | 13 | 0 | 0 | 0 | NA |
| C19.0-C21.9 | 49 | 17 | 0 | 0 | 0 | NA |
| C19.0-C21.9 | 59 | 102 | 0 | 0 | 0 | NA |
| C19.0-C21.9 | 65 | 870 | 12 | 6 | 0.37 | 8.00E-02 |
| C19.0-C21.9 | 73 | 1160 | 21 | 5 | 0.23 | 4.80E-01 |
| C19.0-C21.9 | 77 | 576 | 6 | 4 | 0.38 | 1.52E-01 |
| C19.0-C21.9 | 83 | 565 | 8 | 0 | 0 | NA |
| C19.0-C21.9 | 84 | 8 | 0 | 0 | 0 | NA |
| C19.0-C21.9 | 94 | 8 | 0 | 0 | 0 | NA |
| C19.0-C21.9 | 97 | 2 | 0 | 0 | 0 | NA |
| C19.0-C21.9 | 99 | 602 | 4 | 2 | 0.18 | 7.78E-01 |
| C22.0-C22.1 | 4 | 580 | 2 | 2 | 0.19 | 7.78E-01 |
| C22.0-C22.1 | 15 | 1605 | 41 | 13 | 0.44 | 3.75E-03 |
| C22.0-C22.1 | 23 | 5 | 0 | 0 | 0 | NA |
| C22.0-C22.1 | 24 | 4 | 0 | 0 | 0 | NA |
| C22.0-C22.1 | 28 | 526 | 4 | 2 | 0.21 | 7.69E-01 |
| C22.0-C22.1 | 29 | 7 | 0 | 0 | 0 | NA |
| C22.0-C22.1 | 33 | 87 | 0 | 0 | 0 | NA |
| C22.0-C22.1 | 44 | 2 | 0 | 0 | 0 | NA |
| C22.0-C22.1 | 45 | 48 | 2 | 2 | 2.25 | 2.26E-02 |
| C22.0-C22.1 | 46 | 4887 | 246 | 30 | 0.33 | 9.96E-04 |
| C22.0-C22.1 | 49 | 7 | 0 | 0 | 0 | NA |
| C22.0-C22.1 | 65 | 3 | 0 | 0 | 0 | NA |
| C22.0-C22.1 | 73 | 1997 | 43 | 12 | 0.32 | 3.98E-02 |
| C22.0-C22.1 | 77 | 43 | 0 | 0 | 0 | NA |
| C22.0-C22.1 | 94 | 70 | 2 | 2 | 1.54 | 3.90E-02 |
| C22.0-C22.1 | 99 | 2 | 0 | 0 | 0 | NA |
| C23.9-C24.9 | 4 | 5760 | 266 | 28 | 0.26 | 2.43E-02 |
| C23.9-C24.9 | 6 | 6 | 0 | 0 | 0 | NA |
| C23.9-C24.9 | 14 | 6 | 0 | 0 | 0 | NA |
| C23.9-C24.9 | 15 | 334 | 0 | 0 | 0 | NA |
| C23.9-C24.9 | 23 | 2 | 0 | 0 | 0 | NA |
| C23.9-C24.9 | 24 | 47 | 0 | 0 | 0 | NA |
| C23.9-C24.9 | 28 | 1017 | 14 | 2 | 0.11 | 1.00E+00 |
| C23.9-C24.9 | 29 | 2 | 0 | 0 | 0 | NA |
| C23.9-C24.9 | 33 | 6 | 0 | 0 | 0 | NA |
| C23.9-C24.9 | 49 | 3 | 0 | 0 | 0 | NA |
| C23.9-C24.9 | 65 | 137 | 0 | 0 | 0 | NA |
| C23.9-C24.9 | 73 | 2283 | 63 | 10 | 0.24 | 2.45E-01 |
| C23.9-C24.9 | 77 | 28 | 0 | 0 | 0 | NA |
| C23.9-C24.9 | 83 | 178 | 2 | 2 | 0.61 | 1.59E-01 |
| C23.9-C24.9 | 84 | 6 | 0 | 0 | 0 | NA |
| C23.9-C24.9 | 94 | 6 | 0 | 0 | 0 | NA |
| C23.9-C24.9 | 97 | 4 | 0 | 0 | 0 | NA |
| C23.9-C24.9 | 99 | 92 | 0 | 0 | 0 | NA |
| C25.0-C25.9 | 1 | 21 | 0 | 0 | 0 | NA |
| C25.0-C25.9 | 4 | 12022 | 788 | 40 | 0.18 | 4.78E-01 |
| C25.0-C25.9 | 6 | 6 | 0 | 0 | 0 | NA |
| C25.0-C25.9 | 14 | 2 | 0 | 0 | 0 | NA |
| C25.0-C25.9 | 23 | 43 | 0 | 0 | 0 | NA |
| C25.0-C25.9 | 24 | 930 | 24 | 12 | 0.7 | 1.43E-04 |
| C25.0-C25.9 | 28 | 2733 | 57 | 14 | 0.28 | 8.47E-02 |
| C25.0-C25.9 | 29 | 7 | 0 | 0 | 0 | NA |
| C25.0-C25.9 | 33 | 47 | 0 | 0 | 0 | NA |
| C25.0-C25.9 | 49 | 7 | 0 | 0 | 0 | NA |
| C25.0-C25.9 | 65 | 361 | 0 | 0 | 0 | NA |
| C25.0-C25.9 | 73 | 11365 | 814 | 29 | 0.14 | 6.41E-01 |
| C25.0-C25.9 | 77 | 386 | 10 | 10 | 1.4 | 2.75E-06 |
| C25.0-C25.9 | 83 | 58 | 0 | 0 | 0 | NA |
| C25.0-C25.9 | 94 | 5 | 0 | 0 | 0 | NA |
| C25.0-C25.9 | 96 | 4 | 0 | 0 | 0 | NA |
| C25.0-C25.9 | 97 | 8 | 0 | 0 | 0 | NA |
| C25.0-C25.9 | 99 | 54 | 0 | 0 | 0 | NA |
| C26.8-C26.9 | 4 | 502 | 4 | 2 | 0.22 | 7.69E-01 |
| C26.8-C26.9 | 28 | 118 | 0 | 0 | 0 | NA |
| C26.8-C26.9 | 33 | 9 | 0 | 0 | 0 | NA |
| C26.8-C26.9 | 34 | 24 | 0 | 0 | 0 | NA |
| C26.8-C26.9 | 65 | 22 | 0 | 0 | 0 | NA |
| C26.8-C26.9 | 73 | 1905 | 84 | 12 | 0.34 | 3.50E-02 |
| C26.8-C26.9 | 77 | 40 | 0 | 0 | 0 | NA |
| C26.8-C26.9 | 83 | 2 | 0 | 0 | 0 | NA |
| C26.8-C26.9 | 99 | 2 | 0 | 0 | 0 | NA |
| C30.0-C31.9 | 4 | 91 | 0 | 0 | 0 | NA |
| C30.0-C31.9 | 5 | 71 | 0 | 0 | 0 | NA |
| C30.0-C31.9 | 10 | 2 | 0 | 0 | 0 | NA |
| C30.0-C31.9 | 16 | 5 | 0 | 0 | 0 | NA |
| C30.0-C31.9 | 17 | 3 | 0 | 0 | 0 | NA |
| C30.0-C31.9 | 28 | 154 | 4 | 4 | 1.4 | 3.93E-03 |
| C30.0-C31.9 | 29 | 26 | 0 | 0 | 0 | NA |
| C30.0-C31.9 | 33 | 187 | 0 | 0 | 0 | NA |
| C30.0-C31.9 | 49 | 4 | 0 | 0 | 0 | NA |
| C30.0-C31.9 | 55 | 2 | 0 | 0 | 0 | NA |
| C30.0-C31.9 | 59 | 161 | 0 | 0 | 0 | NA |
| C30.0-C31.9 | 64 | 2 | 0 | 0 | 0 | NA |
| C30.0-C31.9 | 65 | 11 | 0 | 0 | 0 | NA |
| C30.0-C31.9 | 67 | 18 | 0 | 0 | 0 | NA |
| C30.0-C31.9 | 73 | 65 | 0 | 0 | 0 | NA |
| C30.0-C31.9 | 76 | 35 | 0 | 0 | 0 | NA |
| C30.0-C31.9 | 77 | 14 | 0 | 0 | 0 | NA |
| C30.0-C31.9 | 83 | 13 | 0 | 0 | 0 | NA |
| C30.0-C31.9 | 84 | 2 | 0 | 0 | 0 | NA |
| C30.0-C31.9 | 85 | 2 | 0 | 0 | 0 | NA |
| C30.0-C31.9 | 93 | 15 | 0 | 0 | 0 | NA |
| C30.0-C31.9 | 94 | 18 | 0 | 0 | 0 | NA |
| C30.0-C31.9 | 97 | 2 | 0 | 0 | 0 | NA |
| C30.0-C31.9 | 99 | 742 | 8 | 2 | 0.15 | 1.00E+00 |
| C30.0-C31.9 | 102 | 60 | 0 | 0 | 0 | NA |
| C32.0-C32.9 | 4 | 15 | 0 | 0 | 0 | NA |
| C32.0-C32.9 | 5 | 4 | 0 | 0 | 0 | NA |
| C32.0-C32.9 | 16 | 20 | 0 | 0 | 0 | NA |
| C32.0-C32.9 | 28 | 209 | 0 | 0 | 0 | NA |
| C32.0-C32.9 | 29 | 3 | 0 | 0 | 0 | NA |
| C32.0-C32.9 | 33 | 18 | 0 | 0 | 0 | NA |
| C32.0-C32.9 | 73 | 105 | 0 | 0 | 0 | NA |
| C32.0-C32.9 | 77 | 6 | 0 | 0 | 0 | NA |
| C32.0-C32.9 | 94 | 5 | 0 | 0 | 0 | NA |
| C32.0-C32.9 | 99 | 5967 | 292 | 14 | 0.13 | 6.41E-01 |
| C32.0-C32.9 | 102 | 2 | 0 | 0 | 0 | NA |
| C34.0-C34.9 | 1 | 5 | 0 | 0 | 0 | NA |
| C34.0-C34.9 | 4 | 13290 | 948 | 39 | 0.16 | 9.88E-01 |
| C34.0-C34.9 | 5 | 53 | 0 | 0 | 0 | NA |
| C34.0-C34.9 | 6 | 39 | 0 | 0 | 0 | NA |
| C34.0-C34.9 | 14 | 21 | 0 | 0 | 0 | NA |
| C34.0-C34.9 | 16 | 7 | 0 | 0 | 0 | NA |
| C34.0-C34.9 | 21 | 2 | 0 | 0 | 0 | NA |
| C34.0-C34.9 | 28 | 9851 | 637 | 26 | 0.14 | 7.83E-01 |
| C34.0-C34.9 | 29 | 33 | 0 | 0 | 0 | NA |
| C34.0-C34.9 | 33 | 243 | 2 | 2 | 0.44 | 2.31E-01 |
| C34.0-C34.9 | 44 | 7 | 0 | 0 | 0 | NA |
| C34.0-C34.9 | 47 | 8 | 0 | 0 | 0 | NA |
| C34.0-C34.9 | 49 | 16 | 0 | 0 | 0 | NA |
| C34.0-C34.9 | 51 | 4 | 0 | 0 | 0 | NA |
| C34.0-C34.9 | 59 | 2 | 0 | 0 | 0 | NA |
| C34.0-C34.9 | 65 | 86 | 0 | 0 | 0 | NA |
| C34.0-C34.9 | 66 | 8 | 0 | 0 | 0 | NA |
| C34.0-C34.9 | 67 | 2 | 0 | 0 | 0 | NA |
| C34.0-C34.9 | 73 | 28176 | 2236 | 24 | 0.05 | 2.02E-12 |
| C34.0-C34.9 | 75 | 2 | 0 | 0 | 0 | NA |
| C34.0-C34.9 | 77 | 807 | 18 | 6 | 0.4 | 6.25E-02 |
| C34.0-C34.9 | 81 | 2 | 0 | 0 | 0 | NA |
| C34.0-C34.9 | 83 | 128 | 0 | 0 | 0 | NA |
| C34.0-C34.9 | 84 | 2 | 0 | 0 | 0 | NA |
| C34.0-C34.9 | 91 | 3 | 0 | 0 | 0 | NA |
| C34.0-C34.9 | 94 | 80 | 0 | 0 | 0 | NA |
| C34.0-C34.9 | 97 | 12743 | 993 | 30 | 0.13 | 3.70E-01 |
| C34.0-C34.9 | 99 | 23880 | 2212 | 18 | 0.04 | 7.87E-12 |
| C34.0-C34.9 | 100 | 14 | 0 | 0 | 0 | NA |
| C37.0-C37.9 | 28 | 10 | 0 | 0 | 0 | NA |
| C37.0-C37.9 | 73 | 23 | 0 | 0 | 0 | NA |
| C37.0-C37.9 | 77 | 9 | 0 | 0 | 0 | NA |
| C37.0-C37.9 | 99 | 12 | 0 | 0 | 0 | NA |
| C37.0-C37.9 | 101 | 182 | 2 | 2 | 0.59 | 1.62E-01 |
| C38.0-C38.9 | 33 | 3 | 0 | 0 | 0 | NA |
| C38.0-C38.9 | 49 | 2 | 0 | 0 | 0 | NA |
| C38.0-C38.9 | 63 | 2 | 0 | 0 | 0 | NA |
| C38.0-C38.9 | 100 | 2 | 0 | 0 | 0 | NA |
| C38.1-C38.3,C38.8 | 4 | 3 | 0 | 0 | 0 | NA |
| C38.1-C38.3,C38.8 | 13 | 2 | 0 | 0 | 0 | NA |
| C38.1-C38.3,C38.8 | 28 | 19 | 0 | 0 | 0 | NA |
| C38.1-C38.3,C38.8 | 29 | 6 | 0 | 0 | 0 | NA |
| C38.1-C38.3,C38.8 | 33 | 55 | 0 | 0 | 0 | NA |
| C38.1-C38.3,C38.8 | 35 | 24 | 0 | 0 | 0 | NA |
| C38.1-C38.3,C38.8 | 36 | 12 | 0 | 0 | 0 | NA |
| C38.1-C38.3,C38.8 | 37 | 7 | 0 | 0 | 0 | NA |
| C38.1-C38.3,C38.8 | 38 | 2 | 0 | 0 | 0 | NA |
| C38.1-C38.3,C38.8 | 39 | 2 | 0 | 0 | 0 | NA |
| C38.1-C38.3,C38.8 | 47 | 12 | 0 | 0 | 0 | NA |
| C38.1-C38.3,C38.8 | 49 | 2 | 0 | 0 | 0 | NA |
| C38.1-C38.3,C38.8 | 51 | 4 | 0 | 0 | 0 | NA |
| C38.1-C38.3,C38.8 | 55 | 4 | 0 | 0 | 0 | NA |
| C38.1-C38.3,C38.8 | 73 | 194 | 0 | 0 | 0 | NA |
| C38.1-C38.3,C38.8 | 77 | 19 | 0 | 0 | 0 | NA |
| C38.1-C38.3,C38.8 | 85 | 2 | 0 | 0 | 0 | NA |
| C38.1-C38.3,C38.8 | 89 | 4 | 0 | 0 | 0 | NA |
| C38.1-C38.3,C38.8 | 94 | 3 | 0 | 0 | 0 | NA |
| C38.1-C38.3,C38.8 | 97 | 8 | 0 | 0 | 0 | NA |
| C38.1-C38.3,C38.8 | 99 | 7 | 0 | 0 | 0 | NA |
| C38.4 | 4 | 5 | 0 | 0 | 0 | NA |
| C38.4 | 14 | 2 | 0 | 0 | 0 | NA |
| C38.4 | 28 | 4 | 0 | 0 | 0 | NA |
| C38.4 | 29 | 14 | 0 | 0 | 0 | NA |
| C38.4 | 33 | 16 | 0 | 0 | 0 | NA |
| C38.4 | 63 | 1750 | 72 | 20 | 0.62 | 3.49E-06 |
| C38.4 | 73 | 251 | 0 | 0 | 0 | NA |
| C38.4 | 94 | 17 | 0 | 0 | 0 | NA |
| C39.0-C39.9 | 73 | 22 | 0 | 0 | 0 | NA |
| C40.0-C41.9 | 8 | 134 | 0 | 0 | 0 | NA |
| C40.0-C41.9 | 16 | 662 | 8 | 2 | 0.16 | 7.83E-01 |
| C40.0-C41.9 | 17 | 127 | 0 | 0 | 0 | NA |
| C40.0-C41.9 | 28 | 4 | 0 | 0 | 0 | NA |
| C40.0-C41.9 | 29 | 43 | 0 | 0 | 0 | NA |
| C40.0-C41.9 | 30 | 141 | 0 | 0 | 0 | NA |
| C40.0-C41.9 | 33 | 166 | 0 | 0 | 0 | NA |
| C40.0-C41.9 | 37 | 9 | 0 | 0 | 0 | NA |
| C40.0-C41.9 | 44 | 2 | 0 | 0 | 0 | NA |
| C40.0-C41.9 | 49 | 4 | 0 | 0 | 0 | NA |
| C40.0-C41.9 | 67 | 32 | 0 | 0 | 0 | NA |
| C40.0-C41.9 | 73 | 151 | 0 | 0 | 0 | NA |
| C40.0-C41.9 | 81 | 669 | 16 | 4 | 0.32 | 2.01E-01 |
| C40.0-C41.9 | 94 | 175 | 0 | 0 | 0 | NA |
| C42.0-C42.4 | 2 | 589 | 6 | 2 | 0.18 | 7.78E-01 |
| C42.0-C42.4 | 3 | 5399 | 237 | 24 | 0.24 | 7.82E-02 |
| C42.0-C42.4 | 12 | 7478 | 610 | 66 | 0.48 | 3.37E-13 |
| C42.0-C42.4 | 13 | 8 | 0 | 0 | 0 | NA |
| C42.0-C42.4 | 19 | 2276 | 64 | 17 | 0.4 | 1.91E-03 |
| C42.0-C42.4 | 20 | 241 | 0 | 0 | 0 | NA |
| C42.0-C42.4 | 33 | 67 | 0 | 0 | 0 | NA |
| C42.0-C42.4 | 47 | 4 | 0 | 0 | 0 | NA |
| C42.0-C42.4 | 50 | 447 | 0 | 0 | 0 | NA |
| C42.0-C42.4 | 53 | 261 | 2 | 2 | 0.41 | 2.46E-01 |
| C42.0-C42.4 | 56 | 3 | 0 | 0 | 0 | NA |
| C42.0-C42.4 | 67 | 10019 | 677 | 46 | 0.25 | 1.05E-02 |
| C42.0-C42.4 | 69 | 280 | 0 | 0 | 0 | NA |
| C42.0-C42.4 | 70 | 717 | 4 | 2 | 0.15 | 1.00E+00 |
| C42.0-C42.4 | 71 | 2186 | 73 | 28 | 0.69 | 2.75E-09 |
| C42.0-C42.4 | 73 | 3 | 0 | 0 | 0 | NA |
| C42.0-C42.4 | 87 | 2173 | 68 | 20 | 0.5 | 6.07E-05 |
| C42.0-C42.4 | 88 | 2679 | 86 | 20 | 0.4 | 7.56E-04 |
| C42.0-C42.4 | 103 | 413 | 8 | 8 | 1.05 | 1.87E-04 |
| C44.0-C44.9 | 4 | 26 | 0 | 0 | 0 | NA |
| C44.0-C44.9 | 5 | 42 | 0 | 0 | 0 | NA |
| C44.0-C44.9 | 7 | 402 | 2 | 0 | 0 | NA |
| C44.0-C44.9 | 10 | 145725 | 16203 | 29 | 0.01 | 1.62E-136 |
| C44.0-C44.9 | 14 | 2 | 0 | 0 | 0 | NA |
| C44.0-C44.9 | 24 | 3 | 0 | 0 | 0 | NA |
| C44.0-C44.9 | 28 | 257 | 4 | 4 | 0.84 | 1.86E-02 |
| C44.0-C44.9 | 29 | 896 | 14 | 10 | 0.6 | 1.44E-03 |
| C44.0-C44.9 | 33 | 611 | 6 | 4 | 0.35 | 1.66E-01 |
| C44.0-C44.9 | 44 | 9 | 0 | 0 | 0 | NA |
| C44.0-C44.9 | 48 | 537 | 23 | 19 | 1.91 | 2.05E-13 |
| C44.0-C44.9 | 49 | 95 | 0 | 0 | 0 | NA |
| C44.0-C44.9 | 51 | 5 | 0 | 0 | 0 | NA |
| C44.0-C44.9 | 55 | 62 | 0 | 0 | 0 | NA |
| C44.0-C44.9 | 59 | 20931 | 1703 | 86 | 0.22 | 6.16E-03 |
| C44.0-C44.9 | 61 | 326 | 2 | 2 | 0.33 | 3.25E-01 |
| C44.0-C44.9 | 64 | 7 | 0 | 0 | 0 | NA |
| C44.0-C44.9 | 65 | 16 | 0 | 0 | 0 | NA |
| C44.0-C44.9 | 67 | 2 | 0 | 0 | 0 | NA |
| C44.0-C44.9 | 68 | 578 | 2 | 0 | 0 | NA |
| C44.0-C44.9 | 73 | 255 | 2 | 2 | 0.42 | 2.45E-01 |
| C44.0-C44.9 | 75 | 3 | 0 | 0 | 0 | NA |
| C44.0-C44.9 | 82 | 28 | 0 | 0 | 0 | NA |
| C44.0-C44.9 | 83 | 3 | 0 | 0 | 0 | NA |
| C44.0-C44.9 | 84 | 21 | 0 | 0 | 0 | NA |
| C44.0-C44.9 | 94 | 91 | 0 | 0 | 0 | NA |
| C44.0-C44.9 | 99 | 21757 | 1930 | 64 | 0.16 | 9.56E-01 |
| C47.0-C47.9 | 73 | 3 | 0 | 0 | 0 | NA |
| C47.0-C47.9 | 75 | 264 | 0 | 0 | 0 | NA |
| C47.0-C47.9 | 76 | 420 | 2 | 2 | 0.26 | 4.34E-01 |
| C47.0-C47.9 | 85 | 16 | 0 | 0 | 0 | NA |
| C48.0-C48.8 | 4 | 11 | 0 | 0 | 0 | NA |
| C48.0-C48.8 | 13 | 3 | 0 | 0 | 0 | NA |
| C48.0-C48.8 | 28 | 4 | 0 | 0 | 0 | NA |
| C48.0-C48.8 | 33 | 46 | 0 | 0 | 0 | NA |
| C48.0-C48.8 | 34 | 8 | 0 | 0 | 0 | NA |
| C48.0-C48.8 | 49 | 18 | 0 | 0 | 0 | NA |
| C48.0-C48.8 | 51 | 36 | 0 | 0 | 0 | NA |
| C48.0-C48.8 | 63 | 258 | 2 | 2 | 0.42 | 2.45E-01 |
| C48.0-C48.8 | 65 | 6 | 0 | 0 | 0 | NA |
| C48.0-C48.8 | 73 | 34 | 0 | 0 | 0 | NA |
| C48.0-C48.8 | 77 | 6 | 0 | 0 | 0 | NA |
| C48.0-C48.8 | 94 | 6 | 0 | 0 | 0 | NA |
| C48.0-C48.8 | 96 | 103 | 0 | 0 | 0 | NA |
| C49.0-C49.9 | 13 | 2 | 0 | 0 | 0 | NA |
| C49.0-C49.9 | 14 | 4 | 0 | 0 | 0 | NA |
| C49.0-C49.9 | 16 | 55 | 0 | 0 | 0 | NA |
| C49.0-C49.9 | 17 | 3 | 0 | 0 | 0 | NA |
| C49.0-C49.9 | 28 | 3 | 0 | 0 | 0 | NA |
| C49.0-C49.9 | 29 | 1694 | 33 | 13 | 0.41 | 5.54E-03 |
| C49.0-C49.9 | 30 | 34 | 0 | 0 | 0 | NA |
| C49.0-C49.9 | 33 | 283 | 2 | 2 | 0.38 | 2.71E-01 |
| C49.0-C49.9 | 34 | 41 | 0 | 0 | 0 | NA |
| C49.0-C49.9 | 35 | 2 | 0 | 0 | 0 | NA |
| C49.0-C49.9 | 36 | 12 | 0 | 0 | 0 | NA |
| C49.0-C49.9 | 37 | 29 | 0 | 0 | 0 | NA |
| C49.0-C49.9 | 41 | 2 | 0 | 0 | 0 | NA |
| C49.0-C49.9 | 44 | 76 | 2 | 2 | 1.42 | 4.38E-02 |
| C49.0-C49.9 | 47 | 2 | 0 | 0 | 0 | NA |
| C49.0-C49.9 | 48 | 3 | 0 | 0 | 0 | NA |
| C49.0-C49.9 | 49 | 649 | 10 | 8 | 0.67 | 2.69E-03 |
| C49.0-C49.9 | 51 | 1030 | 16 | 8 | 0.42 | 2.89E-02 |
| C49.0-C49.9 | 55 | 7 | 0 | 0 | 0 | NA |
| C49.0-C49.9 | 65 | 3 | 0 | 0 | 0 | NA |
| C49.0-C49.9 | 67 | 11 | 0 | 0 | 0 | NA |
| C49.0-C49.9 | 70 | 2 | 0 | 0 | 0 | NA |
| C49.0-C49.9 | 73 | 236 | 0 | 0 | 0 | NA |
| C49.0-C49.9 | 75 | 28 | 0 | 0 | 0 | NA |
| C49.0-C49.9 | 76 | 2 | 0 | 0 | 0 | NA |
| C49.0-C49.9 | 77 | 7 | 0 | 0 | 0 | NA |
| C49.0-C49.9 | 81 | 13 | 0 | 0 | 0 | NA |
| C49.0-C49.9 | 85 | 11 | 0 | 0 | 0 | NA |
| C49.0-C49.9 | 90 | 32 | 0 | 0 | 0 | NA |
| C49.0-C49.9 | 93 | 239 | 0 | 0 | 0 | NA |
| C49.0-C49.9 | 94 | 964 | 10 | 0 | 0 | NA |
| C49.0-C49.9 | 95 | 2 | 0 | 0 | 0 | NA |
| C49.0-C49.9 | 99 | 2 | 0 | 0 | 0 | NA |
| C49.0-C49.9 | 100 | 215 | 0 | 0 | 0 | NA |
| C50.0-C50.9 | 4 | 5252 | 248 | 24 | 0.25 | 7.22E-02 |
| C50.0-C50.9 | 5 | 66 | 0 | 0 | 0 | NA |
| C50.0-C50.9 | 6 | 4 | 0 | 0 | 0 | NA |
| C50.0-C50.9 | 7 | 31 | 0 | 0 | 0 | NA |
| C50.0-C50.9 | 14 | 9 | 0 | 0 | 0 | NA |
| C50.0-C50.9 | 16 | 3 | 0 | 0 | 0 | NA |
| C50.0-C50.9 | 24 | 77228 | 6226 | 8 | 0.01 | 1.73E-81 |
| C50.0-C50.9 | 28 | 10669 | 706 | 40 | 0.2 | 1.66E-01 |
| C50.0-C50.9 | 29 | 30 | 0 | 0 | 0 | NA |
| C50.0-C50.9 | 31 | 161 | 2 | 2 | 0.67 | 1.39E-01 |
| C50.0-C50.9 | 33 | 132 | 2 | 2 | 0.82 | 1.01E-01 |
| C50.0-C50.9 | 49 | 10 | 0 | 0 | 0 | NA |
| C50.0-C50.9 | 51 | 11 | 0 | 0 | 0 | NA |
| C50.0-C50.9 | 52 | 12553 | 870 | 34 | 0.15 | 8.65E-01 |
| C50.0-C50.9 | 57 | 1268 | 29 | 7 | 0.3 | 1.66E-01 |
| C50.0-C50.9 | 65 | 2564 | 104 | 27 | 0.57 | 2.45E-07 |
| C50.0-C50.9 | 67 | 2 | 0 | 0 | 0 | NA |
| C50.0-C50.9 | 73 | 2123 | 66 | 4 | 0.1 | 6.41E-01 |
| C50.0-C50.9 | 77 | 55 | 0 | 0 | 0 | NA |
| C50.0-C50.9 | 81 | 6 | 0 | 0 | 0 | NA |
| C50.0-C50.9 | 82 | 68 | 0 | 0 | 0 | NA |
| C50.0-C50.9 | 93 | 2 | 0 | 0 | 0 | NA |
| C50.0-C50.9 | 94 | 61 | 0 | 0 | 0 | NA |
| C50.0-C50.9 | 95 | 6 | 0 | 0 | 0 | NA |
| C50.0-C50.9 | 97 | 3 | 0 | 0 | 0 | NA |
| C50.0-C50.9 | 99 | 107 | 0 | 0 | 0 | NA |
| C51.0-C52.9 | 4 | 70 | 0 | 0 | 0 | NA |
| C51.0-C52.9 | 5 | 5 | 0 | 0 | 0 | NA |
| C51.0-C52.9 | 7 | 3 | 0 | 0 | 0 | NA |
| C51.0-C52.9 | 10 | 34 | 0 | 0 | 0 | NA |
| C51.0-C52.9 | 14 | 3 | 0 | 0 | 0 | NA |
| C51.0-C52.9 | 21 | 5 | 0 | 0 | 0 | NA |
| C51.0-C52.9 | 26 | 5 | 0 | 0 | 0 | NA |
| C51.0-C52.9 | 28 | 143 | 0 | 0 | 0 | NA |
| C51.0-C52.9 | 29 | 8 | 0 | 0 | 0 | NA |
| C51.0-C52.9 | 33 | 16 | 0 | 0 | 0 | NA |
| C51.0-C52.9 | 36 | 2 | 0 | 0 | 0 | NA |
| C51.0-C52.9 | 44 | 3 | 0 | 0 | 0 | NA |
| C51.0-C52.9 | 49 | 21 | 0 | 0 | 0 | NA |
| C51.0-C52.9 | 59 | 163 | 0 | 0 | 0 | NA |
| C51.0-C52.9 | 65 | 2 | 0 | 0 | 0 | NA |
| C51.0-C52.9 | 73 | 80 | 0 | 0 | 0 | NA |
| C51.0-C52.9 | 82 | 59 | 0 | 0 | 0 | NA |
| C51.0-C52.9 | 83 | 7 | 0 | 0 | 0 | NA |
| C51.0-C52.9 | 84 | 6 | 0 | 0 | 0 | NA |
| C51.0-C52.9 | 93 | 2 | 0 | 0 | 0 | NA |
| C51.0-C52.9 | 94 | 29 | 0 | 0 | 0 | NA |
| C51.0-C52.9 | 99 | 3092 | 133 | 36 | 0.63 | 1.37E-10 |
| C53.0-C53.8 | 4 | 84 | 0 | 0 | 0 | NA |
| C53.0-C53.8 | 6 | 5 | 0 | 0 | 0 | NA |
| C53.0-C53.8 | 14 | 2 | 0 | 0 | 0 | NA |
| C53.0-C53.8 | 26 | 22 | 0 | 0 | 0 | NA |
| C53.0-C53.8 | 28 | 36 | 0 | 0 | 0 | NA |
| C53.0-C53.8 | 73 | 4 | 0 | 0 | 0 | NA |
| C53.0-C53.8 | 99 | 145 | 0 | 0 | 0 | NA |
| C53.9-C54.9 | 4 | 2219 | 56 | 6 | 0.15 | 1.00E+00 |
| C53.9-C54.9 | 5 | 4 | 0 | 0 | 0 | NA |
| C53.9-C54.9 | 6 | 26 | 0 | 0 | 0 | NA |
| C53.9-C54.9 | 10 | 3 | 0 | 0 | 0 | NA |
| C53.9-C54.9 | 14 | 416 | 4 | 2 | 0.26 | 4.32E-01 |
| C53.9-C54.9 | 21 | 58 | 0 | 0 | 0 | NA |
| C53.9-C54.9 | 26 | 21410 | 1537 | 32 | 0.08 | 2.22E-04 |
| C53.9-C54.9 | 28 | 1452 | 26 | 12 | 0.45 | 4.65E-03 |
| C53.9-C54.9 | 29 | 3 | 0 | 0 | 0 | NA |
| C53.9-C54.9 | 33 | 33 | 0 | 0 | 0 | NA |
| C53.9-C54.9 | 36 | 2 | 0 | 0 | 0 | NA |
| C53.9-C54.9 | 39 | 73 | 0 | 0 | 0 | NA |
| C53.9-C54.9 | 49 | 528 | 2 | 2 | 0.2 | 7.69E-01 |
| C53.9-C54.9 | 51 | 3 | 0 | 0 | 0 | NA |
| C53.9-C54.9 | 62 | 23 | 0 | 0 | 0 | NA |
| C53.9-C54.9 | 64 | 2 | 0 | 0 | 0 | NA |
| C53.9-C54.9 | 65 | 48 | 0 | 0 | 0 | NA |
| C53.9-C54.9 | 72 | 8036 | 480 | 24 | 0.16 | 8.94E-01 |
| C53.9-C54.9 | 73 | 614 | 2 | 0 | 0 | NA |
| C53.9-C54.9 | 77 | 12 | 0 | 0 | 0 | NA |
| C53.9-C54.9 | 83 | 965 | 6 | 0 | 0 | NA |
| C53.9-C54.9 | 84 | 3 | 0 | 0 | 0 | NA |
| C53.9-C54.9 | 93 | 10 | 0 | 0 | 0 | NA |
| C53.9-C54.9 | 94 | 262 | 2 | 2 | 0.41 | 2.46E-01 |
| C53.9-C54.9 | 95 | 309 | 0 | 0 | 0 | NA |
| C53.9-C54.9 | 96 | 100 | 2 | 2 | 1.08 | 6.60E-02 |
| C53.9-C54.9 | 97 | 12 | 0 | 0 | 0 | NA |
| C53.9-C54.9 | 99 | 18997 | 1367 | 69 | 0.2 | 1.20E-01 |
| C55.0-C55.9 | 4 | 5 | 0 | 0 | 0 | NA |
| C55.0-C55.9 | 14 | 10 | 0 | 0 | 0 | NA |
| C55.0-C55.9 | 26 | 154 | 0 | 0 | 0 | NA |
| C55.0-C55.9 | 28 | 42 | 0 | 0 | 0 | NA |
| C55.0-C55.9 | 29 | 3 | 0 | 0 | 0 | NA |
| C55.0-C55.9 | 33 | 6 | 0 | 0 | 0 | NA |
| C55.0-C55.9 | 39 | 2 | 0 | 0 | 0 | NA |
| C55.0-C55.9 | 44 | 2 | 0 | 0 | 0 | NA |
| C55.0-C55.9 | 49 | 295 | 2 | 2 | 0.37 | 2.86E-01 |
| C55.0-C55.9 | 73 | 188 | 0 | 0 | 0 | NA |
| C55.0-C55.9 | 77 | 2 | 0 | 0 | 0 | NA |
| C55.0-C55.9 | 83 | 4 | 0 | 0 | 0 | NA |
| C55.0-C55.9 | 93 | 3 | 0 | 0 | 0 | NA |
| C55.0-C55.9 | 94 | 54 | 0 | 0 | 0 | NA |
| C55.0-C55.9 | 95 | 2 | 0 | 0 | 0 | NA |
| C55.0-C55.9 | 99 | 9 | 0 | 0 | 0 | NA |
| C56.0-C56.9 | 4 | 82 | 0 | 0 | 0 | NA |
| C56.0-C56.9 | 14 | 96 | 0 | 0 | 0 | NA |
| C56.0-C56.9 | 21 | 423 | 8 | 6 | 0.77 | 5.44E-03 |
| C56.0-C56.9 | 23 | 640 | 4 | 2 | 0.17 | 7.83E-01 |
| C56.0-C56.9 | 26 | 3311 | 129 | 14 | 0.23 | 2.18E-01 |
| C56.0-C56.9 | 28 | 1135 | 28 | 12 | 0.57 | 7.42E-04 |
| C56.0-C56.9 | 29 | 38 | 0 | 0 | 0 | NA |
| C56.0-C56.9 | 31 | 40 | 0 | 0 | 0 | NA |
| C56.0-C56.9 | 33 | 35 | 0 | 0 | 0 | NA |
| C56.0-C56.9 | 35 | 114 | 0 | 0 | 0 | NA |
| C56.0-C56.9 | 36 | 50 | 0 | 0 | 0 | NA |
| C56.0-C56.9 | 37 | 128 | 0 | 0 | 0 | NA |
| C56.0-C56.9 | 39 | 9 | 0 | 0 | 0 | NA |
| C56.0-C56.9 | 49 | 23 | 0 | 0 | 0 | NA |
| C56.0-C56.9 | 57 | 2 | 0 | 0 | 0 | NA |
| C56.0-C56.9 | 62 | 157 | 0 | 0 | 0 | NA |
| C56.0-C56.9 | 65 | 3674 | 115 | 27 | 0.4 | 1.08E-04 |
| C56.0-C56.9 | 72 | 15 | 0 | 0 | 0 | NA |
| C56.0-C56.9 | 73 | 1416 | 18 | 8 | 0.31 | 1.29E-01 |
| C56.0-C56.9 | 77 | 58 | 0 | 0 | 0 | NA |
| C56.0-C56.9 | 83 | 5 | 0 | 0 | 0 | NA |
| C56.0-C56.9 | 94 | 101 | 0 | 0 | 0 | NA |
| C56.0-C56.9 | 95 | 11 | 0 | 0 | 0 | NA |
| C56.0-C56.9 | 96 | 8175 | 425 | 36 | 0.24 | 3.88E-02 |
| C56.0-C56.9 | 97 | 3 | 0 | 0 | 0 | NA |
| C56.0-C56.9 | 98 | 944 | 29 | 12 | 0.69 | 1.59E-04 |
| C56.0-C56.9 | 99 | 37 | 0 | 0 | 0 | NA |
| C56.0-C56.9 | 100 | 2 | 0 | 0 | 0 | NA |
| C56.0-C56.9 | 102 | 11 | 0 | 0 | 0 | NA |
| C57.0-C57.9 | 4 | 118 | 0 | 0 | 0 | NA |
| C57.0-C57.9 | 14 | 23 | 0 | 0 | 0 | NA |
| C57.0-C57.9 | 21 | 3 | 0 | 0 | 0 | NA |
| C57.0-C57.9 | 23 | 2 | 0 | 0 | 0 | NA |
| C57.0-C57.9 | 26 | 369 | 4 | 4 | 0.59 | 4.86E-02 |
| C57.0-C57.9 | 28 | 129 | 2 | 2 | 0.84 | 9.84E-02 |
| C57.0-C57.9 | 33 | 5 | 0 | 0 | 0 | NA |
| C57.0-C57.9 | 39 | 4 | 0 | 0 | 0 | NA |
| C57.0-C57.9 | 49 | 12 | 0 | 0 | 0 | NA |
| C57.0-C57.9 | 62 | 4 | 0 | 0 | 0 | NA |
| C57.0-C57.9 | 65 | 7 | 0 | 0 | 0 | NA |
| C57.0-C57.9 | 73 | 307 | 0 | 0 | 0 | NA |
| C57.0-C57.9 | 83 | 22 | 0 | 0 | 0 | NA |
| C57.0-C57.9 | 94 | 14 | 0 | 0 | 0 | NA |
| C57.0-C57.9 | 96 | 346 | 2 | 0 | 0 | NA |
| C57.0-C57.9 | 98 | 3 | 0 | 0 | 0 | NA |
| C57.0-C57.9 | 99 | 29 | 0 | 0 | 0 | NA |
| C58.0-C58.9 | 39 | 48 | 0 | 0 | 0 | NA |
| C58.0-C58.9 | 73 | 2 | 0 | 0 | 0 | NA |
| C60.0-C60.9 | 4 | 3 | 0 | 0 | 0 | NA |
| C60.0-C60.9 | 28 | 27 | 0 | 0 | 0 | NA |
| C60.0-C60.9 | 33 | 5 | 0 | 0 | 0 | NA |
| C60.0-C60.9 | 48 | 3 | 0 | 0 | 0 | NA |
| C60.0-C60.9 | 49 | 3 | 0 | 0 | 0 | NA |
| C60.0-C60.9 | 59 | 5 | 0 | 0 | 0 | NA |
| C60.0-C60.9 | 73 | 10 | 0 | 0 | 0 | NA |
| C60.0-C60.9 | 84 | 8 | 0 | 0 | 0 | NA |
| C60.0-C60.9 | 94 | 4 | 0 | 0 | 0 | NA |
| C60.0-C60.9 | 99 | 727 | 6 | 2 | 0.15 | 1.00E+00 |
| C61.0-C69.0 | 1 | 8 | 0 | 0 | 0 | NA |
| C61.0-C69.0 | 4 | 79373 | 7394 | 14 | 0.01 | 2.12E-76 |
| C61.0-C69.0 | 24 | 36 | 0 | 0 | 0 | NA |
| C61.0-C69.0 | 28 | 3187 | 193 | 26 | 0.44 | 2.82E-05 |
| C61.0-C69.0 | 29 | 3 | 0 | 0 | 0 | NA |
| C61.0-C69.0 | 33 | 16 | 0 | 0 | 0 | NA |
| C61.0-C69.0 | 49 | 10 | 0 | 0 | 0 | NA |
| C61.0-C69.0 | 65 | 15 | 0 | 0 | 0 | NA |
| C61.0-C69.0 | 73 | 4602 | 286 | 26 | 0.31 | 6.47E-03 |
| C61.0-C69.0 | 77 | 4 | 0 | 0 | 0 | NA |
| C61.0-C69.0 | 83 | 4 | 0 | 0 | 0 | NA |
| C61.0-C69.0 | 94 | 5 | 0 | 0 | 0 | NA |
| C61.0-C69.0 | 97 | 15 | 0 | 0 | 0 | NA |
| C61.0-C69.0 | 99 | 13 | 0 | 0 | 0 | NA |
| C61.0-C69.0 | 102 | 11 | 0 | 0 | 0 | NA |
| C62.0-C62.9 | 4 | 5 | 0 | 0 | 0 | NA |
| C62.0-C62.9 | 28 | 6 | 0 | 0 | 0 | NA |
| C62.0-C62.9 | 33 | 234 | 0 | 0 | 0 | NA |
| C62.0-C62.9 | 35 | 1478 | 28 | 10 | 0.37 | 3.10E-02 |
| C62.0-C62.9 | 36 | 580 | 4 | 0 | 0 | NA |
| C62.0-C62.9 | 37 | 434 | 2 | 2 | 0.25 | 4.36E-01 |
| C62.0-C62.9 | 38 | 254 | 0 | 0 | 0 | NA |
| C62.0-C62.9 | 39 | 66 | 0 | 0 | 0 | NA |
| C62.0-C62.9 | 49 | 9 | 0 | 0 | 0 | NA |
| C62.0-C62.9 | 51 | 6 | 0 | 0 | 0 | NA |
| C62.0-C62.9 | 73 | 35 | 0 | 0 | 0 | NA |
| C62.0-C62.9 | 77 | 5 | 0 | 0 | 0 | NA |
| C62.0-C62.9 | 83 | 2 | 0 | 0 | 0 | NA |
| C62.0-C62.9 | 93 | 14 | 0 | 0 | 0 | NA |
| C62.0-C62.9 | 94 | 11 | 0 | 0 | 0 | NA |
| C62.0-C62.9 | 98 | 14 | 0 | 0 | 0 | NA |
| C63.0-C63.9 | 4 | 8 | 0 | 0 | 0 | NA |
| C63.0-C63.9 | 10 | 2 | 0 | 0 | 0 | NA |
| C63.0-C63.9 | 28 | 3 | 0 | 0 | 0 | NA |
| C63.0-C63.9 | 29 | 4 | 0 | 0 | 0 | NA |
| C63.0-C63.9 | 33 | 4 | 0 | 0 | 0 | NA |
| C63.0-C63.9 | 49 | 17 | 0 | 0 | 0 | NA |
| C63.0-C63.9 | 51 | 31 | 0 | 0 | 0 | NA |
| C63.0-C63.9 | 59 | 2 | 0 | 0 | 0 | NA |
| C63.0-C63.9 | 63 | 2 | 0 | 0 | 0 | NA |
| C63.0-C63.9 | 73 | 4 | 0 | 0 | 0 | NA |
| C63.0-C63.9 | 82 | 2 | 0 | 0 | 0 | NA |
| C63.0-C63.9 | 93 | 3 | 0 | 0 | 0 | NA |
| C63.0-C63.9 | 94 | 11 | 0 | 0 | 0 | NA |
| C63.0-C63.9 | 99 | 9 | 0 | 0 | 0 | NA |
| C64.0-C64.9 | 4 | 17 | 0 | 0 | 0 | NA |
| C64.0-C64.9 | 21 | 831 | 4 | 0 | 0 | NA |
| C64.0-C64.9 | 28 | 1038 | 8 | 2 | 0.1 | 8.41E-01 |
| C64.0-C64.9 | 29 | 16 | 0 | 0 | 0 | NA |
| C64.0-C64.9 | 33 | 70 | 0 | 0 | 0 | NA |
| C64.0-C64.9 | 49 | 34 | 0 | 0 | 0 | NA |
| C64.0-C64.9 | 51 | 10 | 0 | 0 | 0 | NA |
| C64.0-C64.9 | 73 | 3015 | 104 | 26 | 0.47 | 1.20E-05 |
| C64.0-C64.9 | 74 | 428 | 14 | 10 | 1.26 | 5.98E-06 |
| C64.0-C64.9 | 77 | 15 | 0 | 0 | 0 | NA |
| C64.0-C64.9 | 83 | 383 | 8 | 6 | 0.85 | 3.58E-03 |
| C64.0-C64.9 | 92 | 17318 | 1278 | 39 | 0.12 | 1.85E-01 |
| C64.0-C64.9 | 93 | 7 | 0 | 0 | 0 | NA |
| C64.0-C64.9 | 94 | 26 | 0 | 0 | 0 | NA |
| C64.0-C64.9 | 99 | 43 | 0 | 0 | 0 | NA |
| C64.0-C64.9 | 102 | 1297 | 23 | 6 | 0.25 | 3.73E-01 |
| C66.0-C68.9 | 4 | 243 | 0 | 0 | 0 | NA |
| C66.0-C68.9 | 14 | 11 | 0 | 0 | 0 | NA |
| C66.0-C68.9 | 28 | 813 | 14 | 6 | 0.4 | 6.37E-02 |
| C66.0-C68.9 | 29 | 3 | 0 | 0 | 0 | NA |
| C66.0-C68.9 | 33 | 24 | 0 | 0 | 0 | NA |
| C66.0-C68.9 | 49 | 19 | 0 | 0 | 0 | NA |
| C66.0-C68.9 | 59 | 6 | 0 | 0 | 0 | NA |
| C66.0-C68.9 | 65 | 30 | 0 | 0 | 0 | NA |
| C66.0-C68.9 | 73 | 1137 | 28 | 10 | 0.47 | 6.47E-03 |
| C66.0-C68.9 | 77 | 12 | 0 | 0 | 0 | NA |
| C66.0-C68.9 | 93 | 16 | 0 | 0 | 0 | NA |
| C66.0-C68.9 | 94 | 23 | 0 | 0 | 0 | NA |
| C66.0-C68.9 | 97 | 21 | 0 | 0 | 0 | NA |
| C66.0-C68.9 | 99 | 368 | 4 | 0 | 0 | NA |
| C66.0-C68.9 | 102 | 25758 | 2049 | 38 | 0.08 | 2.75E-05 |
| C69.0-C69.9 | 5 | 7 | 0 | 0 | 0 | NA |
| C69.0-C69.9 | 28 | 11 | 0 | 0 | 0 | NA |
| C69.0-C69.9 | 33 | 153 | 0 | 0 | 0 | NA |
| C69.0-C69.9 | 59 | 1977 | 40 | 16 | 0.44 | 1.23E-03 |
| C69.0-C69.9 | 67 | 3 | 0 | 0 | 0 | NA |
| C69.0-C69.9 | 73 | 123 | 0 | 0 | 0 | NA |
| C69.0-C69.9 | 104 | 206 | 4 | 4 | 1.05 | 9.31E-03 |
| C69.0-C69.9 | 93 | 4 | 0 | 0 | 0 | NA |
| C69.0-C69.9 | 94 | 4 | 0 | 0 | 0 | NA |
| C69.0-C69.9 | 99 | 56 | 0 | 0 | 0 | NA |
| C70.0-C72.9,C75.2 | 9 | 1042 | 29 | 6 | 0.31 | 2.03E-01 |
| C70.0-C72.9,C75.2 | 13 | 2 | 0 | 0 | 0 | NA |
| C70.0-C72.9,C75.2 | 16 | 7 | 0 | 0 | 0 | NA |
| C70.0-C72.9,C75.2 | 17 | 2 | 0 | 0 | 0 | NA |
| C70.0-C72.9,C75.2 | 18 | 71 | 0 | 0 | 0 | NA |
| C70.0-C72.9,C75.2 | 22 | 262 | 0 | 0 | 0 | NA |
| C70.0-C72.9,C75.2 | 25 | 70 | 0 | 0 | 0 | NA |
| C70.0-C72.9,C75.2 | 27 | 586 | 10 | 2 | 0.18 | 7.78E-01 |
| C70.0-C72.9,C75.2 | 29 | 4 | 0 | 0 | 0 | NA |
| C70.0-C72.9,C75.2 | 33 | 660 | 2 | 2 | 0.16 | 7.83E-01 |
| C70.0-C72.9,C75.2 | 35 | 18 | 0 | 0 | 0 | NA |
| C70.0-C72.9,C75.2 | 37 | 17 | 0 | 0 | 0 | NA |
| C70.0-C72.9,C75.2 | 38 | 2 | 0 | 0 | 0 | NA |
| C70.0-C72.9,C75.2 | 40 | 8041 | 371 | 39 | 0.26 | 7.30E-03 |
| C70.0-C72.9,C75.2 | 42 | 141 | 15 | 13 | 4.98 | 2.77E-14 |
| C70.0-C72.9,C75.2 | 43 | 654 | 13 | 6 | 0.5 | 3.17E-02 |
| C70.0-C72.9,C75.2 | 44 | 42 | 0 | 0 | 0 | NA |
| C70.0-C72.9,C75.2 | 51 | 17 | 0 | 0 | 0 | NA |
| C70.0-C72.9,C75.2 | 55 | 7 | 0 | 0 | 0 | NA |
| C70.0-C72.9,C75.2 | 58 | 250 | 2 | 2 | 0.43 | 2.40E-01 |
| C70.0-C72.9,C75.2 | 59 | 30 | 0 | 0 | 0 | NA |
| C70.0-C72.9,C75.2 | 60 | 9515 | 601 | 43 | 0.24 | 1.47E-02 |
| C70.0-C72.9,C75.2 | 67 | 2 | 0 | 0 | 0 | NA |
| C70.0-C72.9,C75.2 | 72 | 1865 | 52 | 8 | 0.23 | 3.58E-01 |
| C70.0-C72.9,C75.2 | 73 | 2346 | 65 | 22 | 0.51 | 1.89E-05 |
| C70.0-C72.9,C75.2 | 75 | 12 | 0 | 0 | 0 | NA |
| C70.0-C72.9,C75.2 | 76 | 14 | 0 | 0 | 0 | NA |
| C70.0-C72.9,C75.2 | 78 | 109 | 0 | 0 | 0 | NA |
| C70.0-C72.9,C75.2 | 79 | 2062 | 56 | 21 | 0.55 | 1.02E-05 |
| C70.0-C72.9,C75.2 | 80 | 272 | 2 | 2 | 0.4 | 2.57E-01 |
| C70.0-C72.9,C75.2 | 81 | 3 | 0 | 0 | 0 | NA |
| C70.0-C72.9,C75.2 | 85 | 18 | 0 | 0 | 0 | NA |
| C70.0-C72.9,C75.2 | 90 | 52 | 0 | 0 | 0 | NA |
| C70.0-C72.9,C75.2 | 94 | 234 | 0 | 0 | 0 | NA |
| C73.9 | 4 | 90 | 0 | 0 | 0 | NA |
| C73.9 | 14 | 2 | 0 | 0 | 0 | NA |
| C73.9 | 28 | 938 | 16 | 4 | 0.23 | 4.36E-01 |
| C73.9 | 29 | 9 | 0 | 0 | 0 | NA |
| C73.9 | 32 | 1824 | 53 | 9 | 0.27 | 1.85E-01 |
| C73.9 | 33 | 237 | 2 | 2 | 0.46 | 2.24E-01 |
| C73.9 | 57 | 335 | 24 | 22 | 3.55 | 7.86E-21 |
| C73.9 | 73 | 397 | 4 | 4 | 0.54 | 5.84E-02 |
| C73.9 | 77 | 5 | 0 | 0 | 0 | NA |
| C73.9 | 83 | 7863 | 556 | 82 | 0.56 | 2.77E-20 |
| C73.9 | 84 | 130 | 0 | 0 | 0 | NA |
| C73.9 | 94 | 12 | 0 | 0 | 0 | NA |
| C73.9 | 99 | 38 | 0 | 0 | 0 | NA |
| C74.0-C74.9 | 4 | 157 | 0 | 0 | 0 | NA |
| C74.0-C74.9 | 28 | 263 | 2 | 2 | 0.41 | 2.46E-01 |
| C74.0-C74.9 | 29 | 2 | 0 | 0 | 0 | NA |
| C74.0-C74.9 | 33 | 17 | 0 | 0 | 0 | NA |
| C74.0-C74.9 | 49 | 2 | 0 | 0 | 0 | NA |
| C74.0-C74.9 | 51 | 2 | 0 | 0 | 0 | NA |
| C74.0-C74.9 | 73 | 125 | 0 | 0 | 0 | NA |
| C74.0-C74.9 | 76 | 23 | 0 | 0 | 0 | NA |
| C74.0-C74.9 | 77 | 7 | 0 | 0 | 0 | NA |
| C74.0-C74.9 | 85 | 184 | 0 | 0 | 0 | NA |
| C75.0 | 4 | 6 | 0 | 0 | 0 | NA |
| C75.0 | 28 | 32 | 0 | 0 | 0 | NA |
| C75.0 | 73 | 4 | 0 | 0 | 0 | NA |
| C75.1 | 4 | 5 | 0 | 0 | 0 | NA |
| C75.1 | 22 | 18 | 0 | 0 | 0 | NA |
| C75.1 | 28 | 3 | 0 | 0 | 0 | NA |
| C75.1 | 35 | 6 | 0 | 0 | 0 | NA |
| C75.1 | 73 | 16 | 0 | 0 | 0 | NA |
| C75.3 | 9 | 4 | 0 | 0 | 0 | NA |
| C75.3 | 27 | 2 | 0 | 0 | 0 | NA |
| C75.3 | 35 | 32 | 0 | 0 | 0 | NA |
| C75.3 | 37 | 2 | 0 | 0 | 0 | NA |
| C75.3 | 38 | 3 | 0 | 0 | 0 | NA |
| C75.3 | 40 | 2 | 0 | 0 | 0 | NA |
| C75.3 | 73 | 16 | 0 | 0 | 0 | NA |
| C75.3 | 86 | 96 | 0 | 0 | 0 | NA |
| C76.0-C76.9,C80.9 | 1 | 2 | 0 | 0 | 0 | NA |
| C76.0-C76.9,C80.9 | 4 | 4653 | 181 | 22 | 0.26 | 5.70E-02 |
| C76.0-C76.9,C80.9 | 5 | 5 | 0 | 0 | 0 | NA |
| C76.0-C76.9,C80.9 | 6 | 2 | 0 | 0 | 0 | NA |
| C76.0-C76.9,C80.9 | 8 | 2 | 0 | 0 | 0 | NA |
| C76.0-C76.9,C80.9 | 13 | 12 | 0 | 0 | 0 | NA |
| C76.0-C76.9,C80.9 | 14 | 18 | 0 | 0 | 0 | NA |
| C76.0-C76.9,C80.9 | 15 | 3 | 0 | 0 | 0 | NA |
| C76.0-C76.9,C80.9 | 16 | 4 | 0 | 0 | 0 | NA |
| C76.0-C76.9,C80.9 | 21 | 4 | 0 | 0 | 0 | NA |
| C76.0-C76.9,C80.9 | 23 | 6 | 0 | 0 | 0 | NA |
| C76.0-C76.9,C80.9 | 24 | 18 | 0 | 0 | 0 | NA |
| C76.0-C76.9,C80.9 | 26 | 3 | 0 | 0 | 0 | NA |
| C76.0-C76.9,C80.9 | 28 | 2573 | 57 | 10 | 0.21 | 4.36E-01 |
| C76.0-C76.9,C80.9 | 29 | 15 | 0 | 0 | 0 | NA |
| C76.0-C76.9,C80.9 | 30 | 2 | 0 | 0 | 0 | NA |
| C76.0-C76.9,C80.9 | 32 | 2 | 0 | 0 | 0 | NA |
| C76.0-C76.9,C80.9 | 33 | 804 | 12 | 2 | 0.13 | 1.00E+00 |
| C76.0-C76.9,C80.9 | 34 | 27 | 0 | 0 | 0 | NA |
| C76.0-C76.9,C80.9 | 35 | 3 | 0 | 0 | 0 | NA |
| C76.0-C76.9,C80.9 | 36 | 15 | 0 | 0 | 0 | NA |
| C76.0-C76.9,C80.9 | 37 | 11 | 0 | 0 | 0 | NA |
| C76.0-C76.9,C80.9 | 39 | 9 | 0 | 0 | 0 | NA |
| C76.0-C76.9,C80.9 | 44 | 13 | 0 | 0 | 0 | NA |
| C76.0-C76.9,C80.9 | 46 | 4 | 0 | 0 | 0 | NA |
| C76.0-C76.9,C80.9 | 47 | 22 | 0 | 0 | 0 | NA |
| C76.0-C76.9,C80.9 | 49 | 43 | 0 | 0 | 0 | NA |
| C76.0-C76.9,C80.9 | 51 | 3 | 0 | 0 | 0 | NA |
| C76.0-C76.9,C80.9 | 52 | 3 | 0 | 0 | 0 | NA |
| C76.0-C76.9,C80.9 | 54 | 2 | 0 | 0 | 0 | NA |
| C76.0-C76.9,C80.9 | 55 | 37 | 0 | 0 | 0 | NA |
| C76.0-C76.9,C80.9 | 56 | 15 | 0 | 0 | 0 | NA |
| C76.0-C76.9,C80.9 | 59 | 30 | 0 | 0 | 0 | NA |
| C76.0-C76.9,C80.9 | 62 | 2 | 0 | 0 | 0 | NA |
| C76.0-C76.9,C80.9 | 63 | 5 | 0 | 0 | 0 | NA |
| C76.0-C76.9,C80.9 | 65 | 415 | 2 | 0 | 0 | NA |
| C76.0-C76.9,C80.9 | 67 | 6 | 0 | 0 | 0 | NA |
| C76.0-C76.9,C80.9 | 70 | 2 | 0 | 0 | 0 | NA |
| C76.0-C76.9,C80.9 | 73 | 10218 | 691 | 24 | 0.13 | 4.36E-01 |
| C76.0-C76.9,C80.9 | 76 | 2 | 0 | 0 | 0 | NA |
| C76.0-C76.9,C80.9 | 77 | 375 | 2 | 2 | 0.29 | 3.79E-01 |
| C76.0-C76.9,C80.9 | 81 | 2 | 0 | 0 | 0 | NA |
| C76.0-C76.9,C80.9 | 83 | 176 | 0 | 0 | 0 | NA |
| C76.0-C76.9,C80.9 | 85 | 6 | 0 | 0 | 0 | NA |
| C76.0-C76.9,C80.9 | 89 | 4 | 0 | 0 | 0 | NA |
| C76.0-C76.9,C80.9 | 90 | 5 | 0 | 0 | 0 | NA |
| C76.0-C76.9,C80.9 | 93 | 10 | 0 | 0 | 0 | NA |
| C76.0-C76.9,C80.9 | 94 | 109 | 0 | 0 | 0 | NA |
| C76.0-C76.9,C80.9 | 96 | 56 | 0 | 0 | 0 | NA |
| C76.0-C76.9,C80.9 | 97 | 40 | 0 | 0 | 0 | NA |
| C76.0-C76.9,C80.9 | 99 | 708 | 10 | 6 | 0.46 | 3.97E-02 |
| C76.0-C76.9,C80.9 | 100 | 19 | 0 | 0 | 0 | NA |
| C76.0-C76.9,C80.9 | 102 | 2 | 0 | 0 | 0 | NA |
| C77.0-C77.9 | 12 | 2 | 0 | 0 | 0 | NA |
| C77.0-C77.9 | 13 | 20 | 0 | 0 | 0 | NA |
| C77.0-C77.9 | 33 | 16921 | 1143 | 29 | 0.09 | 7.60E-03 |
| C77.0-C77.9 | 47 | 5499 | 265 | 47 | 0.46 | 2.77E-09 |
| C77.0-C77.9 | 54 | 2 | 0 | 0 | 0 | NA |
| C77.0-C77.9 | 55 | 151 | 0 | 0 | 0 | NA |
| C77.0-C77.9 | 67 | 3 | 0 | 0 | 0 | NA |
| C77.0-C77.9 | 70 | 2 | 0 | 0 | 0 | NA |
| C77.0-C77.9 | 73 | 19 | 0 | 0 | 0 | NA |
| C77.0-C77.9 | 89 | 13 | 0 | 0 | 0 | NA |
